# Ability and knowledge: from epistemic transition systems to labelled stit models

**DOI:** 10.1007/s10458-024-09661-w

**Published:** 2024-11-06

**Authors:** Alexandra Kuncová, Jan Broersen, Hein Duijf, Aldo Iván Ramírez Abarca

**Affiliations:** 1 Department of Philosophy and Religious Studies, Utrecht University, Utrecht, The Netherlands; 2 Department of Philosophy, Vrije Universiteit Amsterdam, Amsterdam, The Netherlands; 3 Munich Center for Mathematical Philosophy, Ludwig-Maximilians-Universität München, Munich, Germany

**Keywords:** Ability, Knowledge, Modal logic, Multiagent system, Stit theory, Transition system

## Abstract

It is possible to know that one can guarantee a certain result and yet not know how to guarantee it. In such cases one has the ability to guarantee something in a causal sense, but not in an epistemic sense. In this paper we focus on two formalisms used to model both conceptions of ability: one formalism based on epistemic transition systems and the other on labelled stit models. We show a strong correspondence between the two formalisms by providing mappings from the former to the latter for both the languages and the structures. Moreover, we demonstrate that our extension of labelled stit logic is more expressive than the logic of epistemic transition systems.

## Introduction

With the fast-growing world of technical innovations greatly intervening with our daily lives, often expanding our potential in multiple ways, the concept of responsibility has been widely debated [1–3]. In this day and age we can do and we can know more than ever before, but *with great power comes great responsibility*. Under which conditions might one be held responsible? We adopt the commonly recognised intuition that an agent might be held responsible for doing something only if she had the ability to prevent it, but she did not prevent it.^1^ In our investigation we discern two conceptions of ability: ability in a *causal sense* and ability in an *epistemic sense*, where the latter ability is the former ability supported by knowledge (which is not the same as knowledge about having the ability). This distinction can then be passed on to responsibility.

To illustrate two different senses of ability and their implications for the multi-faceted concept of responsibility, consider the following example. Imagine Alice whose Beeble account – Beeble being a cloud where Alice and her colleagues store all their sensitive business data – has been hacked. Beeble has recognised suspicious activity on Alice’s account and has required Alice to verify that she is the rightful owner. To prevent her business data from being abused by the hacker, Alice must sign in from a trusted device and change her password as soon as possible. The trusted device is either her laptop or her remote desktop computer, but she does not know which. Using the wrong device will lock her out for good. In her attempt to verify her rightful ownership, Alice decides to sign in using her remote desktop computer. It turns out that her laptop, not the remote desktop computer, was the trusted device and, as a result, Alice is locked out of her Beeble account for good. The question arises: if her business data gets abused, might Alice be held responsible – or, at least, co-responsible?^2^

The answer depends, among other things, on the sense in which, if any, Alice had the ability to prevent the abuse. Hence, there is a prior question: was Alice able to regain control of her account and prevent the abuse? Again, the answer is not straightforward, it depends, among other things, on the interpretation of the term *ability*. If ability is taken to mean that there exists a course of events initiated by Alice that gives her control of her account, then the answer is yes – we refer to this as having the *causal ability*. After all, Alice could have used her laptop to sign in and, thus, could have prevented the abuse. However, if ability is taken to mean that Alice can *knowingly* see to it that she regains control of her account, then the answer is no – we refer to this as lacking the *epistemic ability*. The distinction is that Alice was able to use either of the two devices, which means that there were two actions available to her and one of them guaranteed that she regained control of her account; however, she did not know which of those two actions actually guaranteed the result. Therefore, Alice had the causal ability to prevent the abuse but lacked the epistemic ability to do so.

We return to our original question: if her business data gets abused, might Alice be held responsible? Having the causal ability implies that Alice is indeed responsible for the abuse in the causal sense. Often times, however, our intuitions about responsibility and blame involve epistemic attitudes and thus the analysis needs to go beyond mere causal abilities.^3^ And since Alice is not epistemically able to regain control of her account, she cannot be held responsible, in this sense, for the abuse of her business data.^4^

To model different senses of responsibility, as indicated by our example, it is vital to explore the distinction between causal and epistemic ability. A better understanding of the concepts of knowledge and ability is thus of high relevance and central importance. Logicians in both philosophy and computer science have developed several formalisations of knowledge, action, ability, and their modes of interaction (for overviews see [5, 6]). In this paper we study two specific frameworks that can be used to model and reason about knowledge and ability and the formal relation between these concepts: *epistemic transition systems* by Naumov and Tao [7] and *labelled stit models* by Horty and Pacuit [8]. We will show that their respective logics are strongly related. Our main result is a careful (and non-trivial) mapping from epistemic transition systems to labelled stit models with a corresponding translation of the logical languages that preserves truth.

Before proceeding, a terminological clarification may be helpful. We use the term *logic* to refer to any triple of a formal language, a class of structures, and a set of compositional evaluation rules, according to which the formulas of that formal language are evaluated on those structures. Even though formal languages play a significant role in this paper, the primary focus is on the classes of structures: on epistemic transition systems [7] and labelled stit models [8]. In the case of epistemic transition systems (in singular abbreviated to an ETS), we refer to their respective logic as *epistemic transition logic* (abbreviated to ETL),^5^ and to their respective formal language as *epistemic transition language* (or as the ETL language). In the case of labelled stit models, we refer to their respective logic as *labelled stit logic* and to their respective formal language as *labelled stit language*.^6^

Specifically, we are most interested in the analysis of *knowing how* by Naumov and Tao [7], on one hand, and the analysis of *epistemic ability* by Horty and Pacuit [8], on the other. We prove that these two terms in fact capture the same concept. In epistemic transition logic, Naumov and Tao characterise knowing how by a single modality of the form $\textsf{H}_i\varphi$, which is to be read as saying that agent *i* knows how to (achieve) $\varphi$. In labelled stit logic, Horty and Pacuit characterise epistemic ability by a complex modality of the form $\Diamond [i\,\textsf{kstit}]\varphi$, which is to be read as saying that agent *i* is epistemically able to see to it that $\varphi$ holds. Our main correspondence result shows that the analysis of knowing how in epistemic transition systems can be simulated by the analysis of epistemic ability in labelled stit models. Moreover, the same holds for the analysis of (one-step) *strategic ability*^7^ by Naumov and Tao and the analysis of *causal ability* by Horty and Pacuit: our main correspondence result shows that the analysis of (one-step) strategic ability in epistemic transition systems can be simulated by the analysis of causal ability in labelled stit models.

There are two main families of logics of action: dynamic logics developed by the community of computer scientists [9] and stit theory developed by the community of philosophers [10].^8^ Where do epistemic transition logic [7] and labelled stit logic [8] fall on this spectrum? Both these logics involve action types in their structures, but not in their formal languages. This separates them from the family of dynamic logics where action types are referred to (by type names) in the formal language. It also separates them from the family of standard stit logics where action types are not involved at all. Therefore, the logics we focus on are a kind of hybrids in between the family of dynamic logics and the family of standard stit logics.^9^

Given that epistemic transition logic [7] is an instance of the family of alternating-time temporal logic, abbreviated to ATL [12], we foresee that our central theorem can be in principle extended to the models of ATL and its epistemic extensions, abbreviated to ATEL [14–18].^10^ The central ability operator in such logics is of the form $\langle \langle i \rangle \rangle \varphi$, which is to be read as saying that agent *i* can ensure $\varphi$, where different logics are proposed for different conceptions of ability. This tradition, including epistemic transition logic, proposes that a given agent is able, in an epistemic sense, to do $\varphi$ if and only if there is an action type available to her that she knows guarantees $\varphi$. Then, in light of our main correspondence result, the analysis of such ability in ATEL models can be in principle simulated by the analysis of epistemic ability in labelled stit models [8].^11^

Our results have prominent implications also for the formal study of moral responsibility. As we have indicated, abilities, notably epistemic abilities, are essential for responsibility attributions. We will show that our extension of labelled stit logic [8] is more expressive than epistemic transition logic [7]. This strongly suggests that the family of stit logic is better suited for the role of a canonical logic for agency and responsibility.

This paper is organised as follows. In Sect. 2 we introduce epistemic transition logic [7] and an extension of labelled stit logic. Our extension of labelled stit logic is presented in several stages: we first present basic stit logic and labelled stit logic [8]; and, then, we present, what we call, *discrete labelled group stit logic* by incorporating several group notions and discrete time. In Sect. 3 we define a language translation mapping epistemic transition language to discrete labelled group stit language. Then, we provide a systematic construction of a labelled stit model based on a given epistemic transition system. With both components – the language translation and the model transformation – we prove our main correspondence result: any epistemic transition system can be transformed into a labelled stit model such that the analysis of knowing how in the former corresponds to that of epistemic ability in the latter. In Sect. 4 we analyse the implications of our correspondence result from the perspective of knowledge representation and reasoning. We show that discrete labelled group stit language is more expressive than epistemic transition language. This further indicates the superiority of stit languages at formalising agency and responsibility. We conclude in Sect. 5.

## Two ways of modelling epistemic ability

In this section we introduce a logic interpreted on epistemic transition systems [7] (2.1) and an extension of a logic interpreted on labelled stit models [8] (2.2).

### Epistemic transition systems

Epistemic transition logic [7] consists of a class of epistemic transition systems and an epistemic transition language. As the main result of their paper, Naumov and Tao represent a sound and complete trimodal axiomatic system for this logic. We, on the other side, study the semantics of this epistemic transition logic.

Following a standard definition, Naumov and Tao [7] characterise epistemic transition systems as labelled directed graphs with vertices denoting states and labelled directed edges (that is, arrows) denoting transitions from one state to another (for classical transition systems see also [22]). These graphs are further supplemented with a set of indistinguishability relations on vertices to capture knowledge, one relation for each agent. Labels on the edges represent the choice that the agents make during the transition. Alternatively, we also talk about the action that the agents perform during the transition.^12^ An epistemic transition system is formally defined as follows.

#### Definition 1

(Epistemic Transition System). An *epistemic transition system* $\mathcal {N}$ is a tuple $\langle \textit{W}$, $\textit{Ags}$, $(\sim _i)$, *V*, $\mathcal {T}$, $\pi \rangle$, where:

- $\textit{W}$ is a nonempty set of states,
- $\textit{Ags}$ is a nonempty finite set of agents,
- $\sim _i\subseteq \textit{W}\times \textit{W}$ is an epistemic equivalence relation, one for each agent $i\in \textit{Ags}$; in particular, $w\sim _i w'$ is taken to mean that the states *w* and $w'$ are epistemically indistinguishable for the agent *i*,
- *V* is a nonempty set of actions available to each agent $i\in \textit{Ags}$ at each state $w\in \textit{W}$; the Cartesian product $V^\textit{Ags}$ is the set of action profiles of $\textit{Ags}$, where each action profile $\textbf{s}\in V^\textit{Ags}$ is a tuple consisting of one action $s_i$ per each agent $i\in \textit{Ags}$, that is, $\textbf{s}=\langle s_1,\dots , s_n\rangle$ for $\textit{Ags}=\{1,\dots ,n\}$, or equivalently, $\textbf{s}=\langle s_i \rangle _{i\in \textit{Ags}}$,^13^
- $\mathcal {T}\subseteq \textit{W}\times V^\textit{Ags}\times \textit{W}$ is a set of transitions, where for each $w\in \textit{W}$ and each $\textbf{s}\in V^\textit{Ags}$ there is $w'\in \textit{W}$ such that $(w,\textbf{s},w')\in \mathcal {T}$; in particular, a triple $(w,\textbf{s},w')\in \mathcal {T}$ represents the transition from state *w* to state $w'$ via action profile $\textbf{s}$,^14^
- $\pi : {\mathcal {P}}\rightarrow 2^\textit{W}$ is a valuation that assigns to each propositional variable $p\in \mathcal {P}$ the set of states $\pi (p)\subseteq W$ where *p* holds.

Further, Naumov and Tao [7] conservatively extend the semantics of ETL to a group setting, where each group notion is defined in terms of its individualistic counterpart. In particular, they define group knowledge in terms of individual knowledge, and group action profiles in terms of individual action profiles. As is standard, we refer to a set of agents $\textit{Ags}$ as the *grand coalition*, and to any group of agents $\textit{C}\subseteq \textit{Ags}$ as a *coalition*.

#### Definition 2

(Knowledge and Action Profiles of Coalitions). Let $\mathcal {N}$ be an epistemic transition system.

- For each coalition $\textit{C}\subseteq \textit{Ags}$ we define
  $$\begin{aligned}\sim _\textit{C}\,{:=}\,\bigcap _{i\in \textit{C}} \sim _i.\end{aligned}$$
  Specifically, for the empty coalition we get $\sim _\emptyset =\textit{W}\times \textit{W}$. Relation $\sim _\textit{C}$ characterises *distributed knowledge* of coalition $\textit{C}$.^15^
- For each coalition $\textit{C}\subseteq \textit{Ags}$ and each action profile $\textbf{s}\in V^\textit{Ags}$ we define
  $$\begin{aligned}\textbf{s}_\textit{C}:= \langle s_i\rangle _{i\in \textit{C}}.\end{aligned}$$
  Specifically, for the empty coalition we get $\textbf{s}_\emptyset =\langle \ \rangle$. We interpret the tuple $\textbf{s}_\textit{C}$ as an action profile of coalition $\textit{C}$. The set of action profiles of coalition $\textit{C}$ is then given by the Cartesian product $V^\textit{C}$.

The ETL language is designed for coalitions, where each individual agent $i\in \textit{Ags}$ is represented by a singleton $\{i\}$.

#### Definition 3

(Syntax). The language $\mathcal {L}_{\textsf {ETL}}$ is defined as follows:

$$\begin{aligned} \varphi \,{:}{:}{=} \ p\mid \lnot \varphi \mid (\varphi \wedge \varphi )\mid \textsf{K}_\textit{C}\varphi \mid \textsf{S}_\textit{C}\varphi \mid \textsf{H}_\textit{C}\varphi , \end{aligned}$$

where *p* ranges over a given countable set of propositional variables $\mathcal {P}$, and $\textit{C}$ ranges over the powerset of a given finite set of agents $\textit{Ags}$.

Naumov and Tao [7] use epistemic transition logic to study a formal language that focuses on three modalities, in particular, knowledge $\textsf{K}$, (one-step) strategic ability $\textsf{S}$, and know-how $\textsf{H}$.^16^ A formula of the form $\textsf{K}_i\varphi$ is to be read as saying that agent *i* knows $\varphi$.^17^ A formula of the form $\textsf{S}_i\varphi$ is to be read as saying that agent *i* has the (one-step) strategic ability to (achieve) $\varphi$, that is, there is an action available to the agent that guarantees $\varphi$. And a formula of the form $\textsf{H}_i\varphi$ is to be read as saying that agent *i* knows how to (achieve) $\varphi$. In terms of epistemic transition logic, an agent knows how to (achieve) $\varphi$ if and only if there is an action available to her that she knows guarantees it. That is, if and only if there is an action available to the agent that achieves $\varphi$ at all states that she cannot epistemically distinguish from the actual state.

In order to provide truth conditions for the modalities in question, we first need to define what it means for a transition to be consistent with a given action profile.

#### Definition 4

(Transitions and Action Profiles). Let $\mathcal {N}$ be an epistemic transition system. Let *w*, $w'\in \textit{W}$ be states, let $\textit{C}\subseteq \textit{Ags}$ be a coalition, and let $\textbf{s}_\textit{C}\in V^\textit{C}$ be an action profile of coalition $\textit{C}$. Then we write $w\overset{\textbf{s}_\textit{C}}{\rightarrow }w'$, if there is an action profile $\mathbf {s'}\in V^\textit{Ags}$ such that $(w,\mathbf {s'},w')\in \mathcal {T}$ and $s'_i=s _i$ for each $i\in \textit{C}$. We say that the transition $(w,\mathbf {s'},w')$ is *consistent* with the action profile $\textbf{s}_\textit{C}$.

In particular, observe that for the unique action profile of the empty coalition $\textbf{s}_\emptyset$, if there are a nonempty coalition $\textit{C}\subseteq \textit{Ags}$ and an action profile $\textbf{s}_\textit{C}$ of coalition $\textit{C}$ such that $w\overset{\textbf{s}_\textit{C}}{\rightarrow }w'$, then $w\overset{\textbf{s}_\emptyset }{\rightarrow }w'$.

#### Definition 5

(Evaluation Rules). Let $\mathcal {N}$ be an epistemic transition system, let $w\in \textit{W}$ be a state, let $\textit{C}\subseteq \textit{Ags}$ be a coalition, and let $\varphi \in \mathcal {L}_{\textsf {ETL}}$ be a formula. Then the truth of $\varphi$ at *w* in $\mathcal {N}$, notation: $\mathcal {N},w\models \varphi$, is given by the following (suppressing the standard propositional clauses):^18^

$$\begin{aligned} \mathcal {N},w\models &   \textsf{K}_\textit{C}\varphi \quad \, \Leftrightarrow \quad \text {for every } w'\in \textit{W}\text { such that } w\sim _\textit{C}w':\mathcal {N},w'\models \varphi ,\\ \mathcal {N},w\models &   \textsf{S}_\textit{C}\varphi \quad \, \Leftrightarrow \quad \text {there is an action profile } \textbf{s}_\textit{C}\in V^\textit{C}\text { such that for every}\\  &   \qquad \qquad \qquad \,\, w'\in \textit{W}:\text { if } w\overset{\textbf{s}_\textit{C}}{\rightarrow }w' \text { then }\mathcal {N},w'\models \varphi ,\\ \mathcal {N},w\models &   \textsf{H}_\textit{C}\varphi \quad \Leftrightarrow \!\quad \, \text {there is an action profile }\textbf{s}_\textit{C}\in V^\textit{C}\text { such that for every}\\  &   \qquad \qquad \quad \,\,\,\,\,\,\,\, w',w''\in \textit{W}:\text { if } w\sim _\textit{C}w'\text { and }w'\overset{\textbf{s}_\textit{C}}{\rightarrow }w''\text { then }\mathcal {N},w''\models \varphi . \end{aligned}$$

To get a better idea of how epistemic transition logic works, it may be helpful to consider how a particular epistemic transition system models certain examples. Let us take the example from the introduction where Alice had the causal ability to regain control of her hacked Beeble account, but lacked the epistemic ability to do so. Consider the moment where Alice is still deliberating about how to regain ownership. To capture this example we make use of four states $w_1$, $w_2$, $u_1$ and $u_2$, where *w*-states stand for the initial states and *u*-states for the end states. In particular, state $w_1$ represents the scenario where the trusted device is the agent’s laptop, state $w_2$ represents the scenario where the trusted device is the agent’s remote desktop computer, state $u_1$ represents the scenario where the agent regains control of her account, whereas state $u_2$ represents the scenario where the agent does not regain control of her account. We take proposition *q* to represent the state of affairs where the agent regains control of her account, and so *q* holds exclusively at $u_1$. At each state the agent has two actions to her disposal: to use her laptop (action $\textsf {L}$) and to use her remote desktop computer (action $\textsf {R}$). From the perspective of state $w_1$, action $\textsf {L}$ leads to state $u_1$ where proposition *q* holds, and action $\textsf {R}$ leads to state $u_2$ where proposition *q* does not hold. From the perspective of state $w_2$, action $\textsf {L}$ leads to state $u_2$ and action $\textsf {R}$ to state $u_1$. The fact that Alice does not know which device is the trusted one is represented by a dashed line labelled *a* between $w_1$ and $w_2$. Notice that there are two different actions available to Alice that guarantee *q*: at $w_1$ it is action $\textsf {L}$, while at $w_2$ it is action $\textsf {R}$, but not vice versa. Thus, at each of these initial states there is an action available to Alice that guarantees *q*. Formally, we have that $w_1\models \textsf{S}_a q$ and $w_2\models \textsf{S}_a q$. Since Alice cannot epistemically distinguish between $w_1$ and $w_2$, it follows that she knows that there is an action available to her that guarantees *q*. Formally, we have that $w_1\models \textsf{K}_a\textsf{S}_aq$. However, since neither action $\textsf {L}$ nor action $\textsf {R}$ guarantees *q* at both initial states, Alice does not know how to achieve *q*. Formally, we have that $w_1\models \lnot \textsf{H}_a q$. The same formulas hold at $w_2$.

**Fig. 1:**
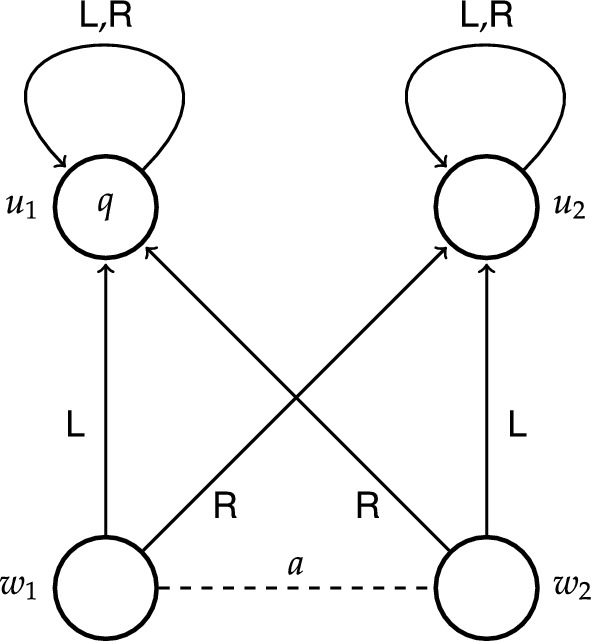
ETS depicting causal ability to *q* at $w_1$

**Fig. 2:**
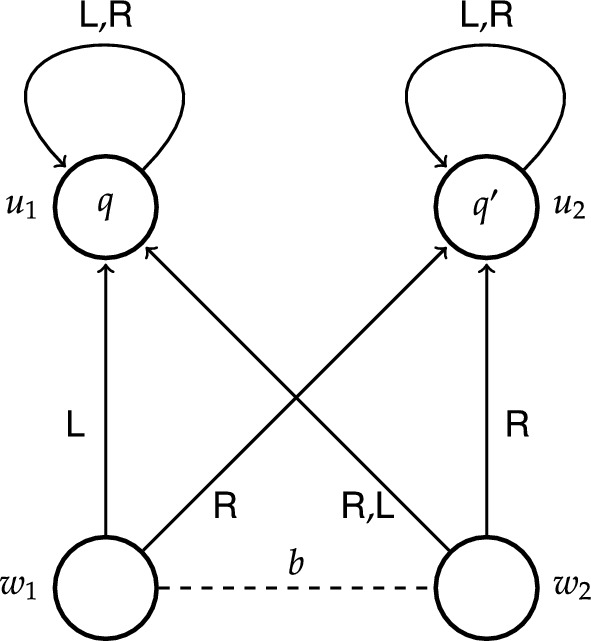
ETS depicting epistemic ability to *q* at $w_1$ and non-determinism of $\textsf {R}$ at $w_2$

We depicted this epistemic transition system in Fig. 1, which illustrates the following conventions: a state $w\in \textit{W}$ is represented by a circle labelled *w*, a transition $(w,\textbf{s},w')\in \mathcal {T}$ is represented by an arrow leading from *w* to $w'$ and labelled $\textbf{s}$, and the fact that a proposition *p* holds at a state *w* is represented by a label *p* placed inside of the circle representing the state *w*. Whenever an agent *i* cannot epistemically distinguish between two states, this is depicted by a dashed line labelled *i* between these states.

Let us look at a slightly different epistemic transition system depicted in Fig. 2 (for the current purpose ignore proposition $q'$ satisfied at state $u_2$). While the model in Fig. 1 was used to illustrate causal ability, this model is used to illustrate epistemic ability and non-determinism. The structural similarity to the former model is that states $w_1$ and $w_2$ cannot be epistemically distinguished, however, there are two important distinctions, both concerning state $w_2$: (i) action $\textsf {L}$ leads to $u_1$, and (ii) action $\textsf {R}$ can lead to either $u_1$ or $u_2$. The latter is depicted by two arrows labelled $\textsf {R}$ starting at $w_2$, one leading to $u_1$, the other to $u_2$. Conceptually, this model might represent, for instance, a situation similar to Alice’s, where her colleague Brox has also had his Beeble account hacked, but unlike Alice, he knows that the trusted device is his laptop. In addition, Brox’ wifi card sometimes malfunctions, and when that happens (state $w_2$) using the remote desktop computer (action $\textsf {R}$) might also lead to success (proposition *q*). Brox knows about this sporadic malfunction, but there is no way for him to recognise the current status of his wifi card; more specifically, Brox cannot distinguish between state $w_1$ representing the scenario where his wifi card works fine and state $w_2$ representing the scenario where his wifi card malfunctions.

As for Brox’ epistemic ability, it is easy to confirm in Fig. 2 that he knows how to regain control of his account. Notice that at both initial states $w_1$ and $w_2$ using his laptop (action $\textsf {L}$) leads exclusively to $u_1$ where *q* holds. Thus, in contrast to Alice (Fig. 1), Brox knows how to achieve *q*, because he knows the specific action that guarantees *q*. Formally, we have that not only $w_1\models \textsf{S}_b q$ and $w_1\models \textsf{K}_b\textsf{S}_b q$, but also $w_1\models \textsf{H}_b q$. The same formulas hold at $w_2$.

In general, epistemic transition systems are very similar to concurrent game structures of ATL [12], specifically, to the structures of ATEL [14–19]. There are, however, a few important distinctions between the two: both in terms of conditions on the structures and in complexity of the semantics [7, p. 12]. First, in epistemic transition systems availability of actions is uniform across states and agents, which means that all actions are available at all states to all agents. One can partially observe this in our single-agent example models in Figs. 1 and 2, where both actions $\textsf {L}$ and $\textsf {R}$ are available at every state, in particular, also at states $u_1$ and $u_2$ (depicted by a reflexive arrow). This rigorous constraint is not imposed on the concurrent game structures of ATL.^19^

Second, in epistemic transition systems actions can be non-deterministic. Let us return to the model in Fig. 2. Notice that there are two arrows with label $\textsf {R}$ starting at $w_2$: one of them leads to $u_1$ and the other one leads to $u_2$. This means that if Brox’ wifi card malfunctions (state $w_2$) while he is using his remote desktop computer (action $\textsf {R}$), the outcome of this action is uncertain, it leads to either $u_1$ or $u_2$. Hence, at $w_2$ Brox does not know how to achieve $q'$, nor there is an action available to him that guarantees $q'$. Formally, we have that $w_2\models \lnot \textsf{H}_b q'$ and $w_2\models \lnot \textsf{S}_b q'$ (yet notice that $w_1\models \textsf{S}_b q'$). The concurrent game structures of ATL cannot represent non-deterministic actions.^20^

Third, the semantics of ETL is based on a more rudimentary notion of strategy than the semantics of ATL. While the former concerns one-step strategies, the latter concerns strategies of an extensive form (that is, multiple-step strategies). To highlight this distinction, in ETL we refer to the one-step strategies simply as actions.

### Labelled stit models

We first introduce basic stit theory (2.2.1), and then cover labelled stit logic [8] (2.2.2). In particular, we extend stit models with knowledge to yield epistemic stit models, and we further extend epistemic stit models with action types to yield labelled stit models. Then, we demonstrate how to use these models to study group notions such as group knowledge, group action, and group ability, and we present the assumption of discrete time (2.2.3). We thus establish, what we call, discrete labelled group stit logic. Finally, we illustrate labelled stit logic by modeling some examples.

#### Stit models

Stit theory grew out of a modal tradition in the logic of action. Its origins go back to early Scandinavian attempts to formalize action as a modality [29, 30]. These were later extended with ideas about agency in branching time by people like Nuel Belnap, Brian Chellas, John Horty, Franz von Kutschera, and many more. Detailed parts of the history and development of stit logics can be found in the book by Nuel Belnap, Michael Perloff and Ming Xu [10].

In the stit tradition, the agency of an individual is characterised by a modal operator of the form $[{i\,\textsf{stit}}]\varphi$, which is to be read as saying that agent *i* *sees to it that* $\varphi$ holds (hence the abbreviation *stit*). In particular, stit semantics is cast against the background of an indeterministic world view, that orders moments in a tree of histories, resulting in a branching-time structure [10, 31]. The forward branching of histories represents the openness of the future. The absence of backward branching represents the determinateness of the past, that is, the fact that every moment has only a single past sequence of events. Each history in such a tree-like structure represents a complete temporal evolution of the world.

##### Definition 6

(Stit Model). A *stit model* $\mathcal {M}$ is a tuple $\langle M$, *H*, <, $\textit{Ags}$, $(\textit{Act}_i^m)$, $\pi \rangle$, where:

- *M* is a nonempty set of moments.
- < is a strict partial ordering on *M* without backward branching: for any $m,m',m''$ in *M*, if $m'<m$ and $m''<m$ then either $m'=m''$, or $m'<m''$, or $m''<m'$.
- $H\subseteq 2^M$ is a set of histories, where a history *h* is a maximal set of linearly ordered moments; in addition, $H_m=\{h\in H\mid m\in h\}$ is the set of histories passing through moment *m*.^21^ We use *m*/*h* as variables for the moment/history pairs that satisfy $h\in H_m$, and we call them *indices* with $\textit{Ind}$ denoting a set of indices.
- $\textit{Ags}$ is a nonempty finite set of agents.
- $\textit{Act}_i^m\subset 2^{H_m}$ is a finite set of actions available to agent $i\in \textit{Ags}$ at moment $m\in M$. $\textit{Act}_i^m$ constitutes a partition of $H_m$. For any $h\in H_m$ we denote by $\textit{Act}_i^m(h)$ the cell of *h* in partition $\textit{Act}_i^m$. These partitions satisfy the following constraints:
  (NC) No Choice between Undivided Histories: for every moment *m*, every agent $i\in \textit{Ags}$, and all histories $h, h'\in H_m$, if there is a moment $m'$ such that $m'\in h\cap h'$ and $m<m'$, then $h\in \textit{Act}^m_i(h')$.
  (IA) Independence of Agency: for every moment *m*, any set of histories $h_i\in H_m$, one for each agent $i\in \textit{Ags}$, it holds that $\bigcap _{i\in \textit{Ags}}\textit{Act}^m_i(h _i)\ne \emptyset$.^22^
- $\pi :\mathcal {P}\rightarrow 2^\textit{Ind}$ is a valuation that assigns to each propositional variable $p\in \mathcal {P}$ the set of indices $\pi (p)\subseteq \textit{Ind}$ where *p* holds.

It is important to note that in stit theory actions are standardly meant as “action tokens – particular, concrete actions, each occurring at a single point in space and time” [8, p. 617]. The particular action that an agent *i* executes at an index *m*/*h* is given by $\textit{Act}^m_i(h)$, which is the action $K\in \textit{Act}^m_i$ satisfying $h\in K$. Hence, each action is identified with a subset of possible histories.

Formula $[{i\,\textsf{stit}}]\varphi$ holds at an index if and only if the truth of $\varphi$ is guaranteed by the action that the agent *i* performs at that index. The idea behind the central agency operator is that the agent *i* performs a certain action and thereby constrains the possible histories to only those where $\varphi$ holds. Phrased differently, she picks one of the partition cells *K* (also called *choice cells*) such that all the histories $h\in K$ satisfy $\varphi$.

##### Definition 7

(Basic Syntax). The language $\mathcal {L}_{\textsf{stit}}$ is defined as follows:

$$\begin{aligned} \varphi \,\,{::=}\ p\mid \lnot \varphi \mid (\varphi \wedge \varphi )\mid \Box \varphi \mid [{i\,\textsf{stit}}]\varphi , \end{aligned}$$

where *p* ranges over a given countable set of propositional variables $\mathcal {P}$, and *i* ranges over a given finite set of agents $\textit{Ags}$. We define $\Diamond \varphi$, in the usual way, as $\lnot \Box \lnot \varphi$, and similarly, $\langle {i\,\textsf{stit}}\rangle \varphi$ as $\lnot [{i\,\textsf{stit}}]\lnot \varphi$.

The historical necessity operator $\Box \varphi$ expresses that $\varphi$ holds regardless of how the future unfolds, alternatively, we say that $\varphi$ is historically settled. Its dual, the historical possibility operator $\Diamond \varphi$ expresses that there is a possible way for the future to unfold such that $\varphi$ holds.

##### Definition 8

(Basic Evaluation Rules). Let $\mathcal {M}$ be a stit model, let *m*/*h* be an index, let $i\in \textit{Ags}$ be an individual agent, and let $\varphi \in \mathcal {L}_{\textsf{stit}}$ be a formula. Then the truth of $\varphi$ at *m*/*h* in $\mathcal {M}$, notation: $\mathcal {M},m/h\models \varphi$, is given by the following (suppressing the standard propositional clauses):

$$\begin{aligned} \mathcal {M},m/h&\models \Box \varphi&\Leftrightarrow \quad&\text {for every } h'\in H_m \text { it holds that } \mathcal {M},m/h'\models \varphi ,\\ \mathcal {M},m/h&\models [{i\,\textsf{stit}}]\varphi&\Leftrightarrow \quad&\text {for every } h'\in \textit{Act}_i^m(h) \text { it holds that } \mathcal {M},m/h'\models \varphi . \end{aligned}$$

Horty and Belnap [32] propose that the ability of an agent can reasonably be characterised by a combination of impersonal (historical) possibility and agency of the form $\Diamond [{i\,\textsf{stit}}]\varphi$, which is to be read as saying that it is possible that agent *i* sees to it that $\varphi$ holds. This notion of ability is taken to stand for *causal* ability. Thus, the formula above can also be read as saying that agent *i* is causally able to $\varphi$. In the following subsection we will introduce *epistemic* ability.

#### Knowledge and action types

Having defined stit theory, we can establish its epistemic extension. In line with the standard modal treatment of knowledge, we supplement stit models with a set of epistemic indistinguishability relations $\sim _i$, one for each agent $i\in \textit{Ags}$.^23^ Recently, Horty and Pacuit [8] have argued that to model epistemic ability, we further need to add action types to epistemic stit models.^24^ This is a substantial addition, because stit theory traditionally focuses not on types of actions, but on particular actions by particular agents at particular moments in time. That is, stit semantics concentrates on action tokens represented by the so called choice cells in the models. Horty and Pacuit claim that “if the epistemic sense of ability requires that some single action must be known by agent *i* to guarantee the truth of $\varphi$, then this must be the action type, not one of its various tokens” [8, p. 626 – notation adapted]. They therefore introduce *labelled* epistemic stit models (often called simply labelled stit models), which are epistemic stit models supplemented with action types. We provide both extensions – adding knowledge and action types to stit models – simultaneously in the following formal definition.

##### Definition 9

(Labelled Stit Model). A  *labelled stit model* $\mathcal {M}$ is a tuple $\langle M$, *H*, <, $\textit{Ags}$, $(\textit{Act}_i^m)$, $(\sim _i)$, $\textit{Tps}$, $\textit{Lbl}$, $\textit{Exn}$, $\pi \rangle$, where:

- $\langle M$, *H*, <, $\textit{Ags}$, $(\textit{Act}_i^m)$, $\pi \rangle$ is a stit model,
- $\sim _i\subseteq \textit{Ind}\times \textit{Ind}$ is an epistemic equivalence relation, one for each agent $i\in \textit{Ags}$; in particular, $m/h\sim _i m'/h'$ is taken to mean that the indices *m*/*h* and $m'/h'$ are epistemically indistinguishable for the agent *i*,
- $\textit{Tps}$ is a set of action types,
- $\textit{Lbl}$ is a label function that maps each action token *K* to a particular action type $\textit{Lbl}(K)\in \textit{Tps}$; we will often write $\textit{Lbl}_i(m/h)$, instead of $\textit{Lbl}(\textit{Act}^m_i(h))$, to denote the action type that agent *i* executes at *m*/*h*,
- $\textit{Exn}$ is a partial execution function that maps each action type $\tau \in \textit{Tps}$, moment *m* and agent *i* to a particular action token $\textit{Exn}^m_i(\tau )\in \textit{Act}^m_i$,^25^
- $\textit{Lbl}$ and $\textit{Exn}$ satisfy the following constraints:
  EL $$\begin{aligned}&\textit{Exn}^m_i(\textit{Lbl} _i(m/h))=\textit{Act}^m_i(h)\text {, and } \end{aligned}$$
  LE $$\begin{aligned}&\text {if }\textit{Exn}^m_i(\tau )\text { is defined, then }\textit{Lbl} _i(\textit{Exn}^m_i(\tau ))=\tau . \end{aligned}$$

The (EL) constraint states that given agent *i*, moment *m* and history *h*, the execution of the action type of an action token yields that action token. Conversely, the (LE) constraint states that given agent *i* and moment *m*, if the execution of an action type by this agent at this moment is defined, then the action type of this execution yields that action type.^26^

Moreover, let us use $\textit{Tps}_i^m$ to denote the set of action types that are available to agent *i* at moment *m* in a given labelled stit model. That is, $\textit{Tps}^m_i=\{\tau \in \textit{Tps}\mid \text { there is an } h\in H_m \text { such that }\textit{Lbl}_i(m/h)=\tau \}$.

In order to capture the epistemic sense of agency of an individual, Horty and Pacuit [8] introduce a new modal operator of the form $[i\,\textsf{kstit}]$, where formula $[i\,\textsf{kstit}]\varphi$ is to be read as saying that agent *i* sees to it that $\varphi$ holds, in an epistemic sense. We say that an agent epistemically sees to it that $\varphi$ holds if and only if she knows that the action type she performs guarantees $\varphi$. Or equivalently, if and only if the action type she performs guarantees $\varphi$ at every epistemically indistinguishable index for her.

Horty and Pacuit [8] further propose to characterise epistemic ability in the same fashion as causal ability, that is, by a combination of impersonal (historical) possibility and epistemic agency of the form $\Diamond [i\,\textsf{kstit}]\varphi$. This formula is to be read as saying that it is possible that agent *i* sees to it that $\varphi$, in an epistemic sense. Or more succinctly, that agent *i* is epistemically able to $\varphi$.

##### Definition 10

(Evaluation Rules Knowledge and Epistemic Agency). Let $\mathcal {M}$ be a labelled stit model, let *m*/*h* be an index, and let $i\in \textit{Ags}$ be an individual agent. Then the evaluation rules for the knowledge operator and the epistemic agency operator at *m*/*h* in $\mathcal {M}$ are given by the following (suppressing the model $\mathcal {M}$):

$$\begin{aligned} m/h&\models \textsf{K}_i\varphi \quad \quad \quad \quad \Leftrightarrow \qquad \! \text {for every }m'/h'\in \textit{Ind}\text { satisfying } m/h\sim _i m'/h'\\&\qquad \quad \qquad \qquad \qquad \qquad \,\,\,\,\! \text {it holds that }m'/h'\models \varphi ,\\ m/h&\models [i\,\textsf{kstit}]\varphi \quad \,\;\! \Leftrightarrow \;\,\,\quad \text {for every } m'/h'\in \textit{Ind} \text{ satisfying } m/h\sim _i m'/h'\text { and for}\\&\qquad \quad \qquad \qquad \quad \quad \quad \quad \,\,\, \text {any } m'/h''\in \textit{Ind}\text { satisfying } \textit{Lbl}_i(m/h)=\textit{Lbl}_i(m'/h'')\\&\qquad \quad \qquad \qquad \quad \quad \quad \quad \,\,\, \text {it holds that }m'/h''\models \varphi . \end{aligned}$$

In order for this definition to make sense, additional constraints have to be imposed on labelled stit frames. Horty and Pacuit [8, p. 628] opt for the following two conditions, for every $m/h,m'/h'\in \textit{Ind}$:

C1 $$\begin{aligned}&\text {If }m/h\sim _i m'/h'\text {, then } \textit{Tps}^m_i=\textit{Tps}^{m'}_i. \end{aligned}$$

C4 $$\begin{aligned}&\text {If }m/h\sim _i m'/h'\text {, then }m/h''\sim _i m'/h'''\text { for all }h''\in H_m\text { and }h'''\in H_{m'}. \end{aligned}$$

Condition (C1) ensures that two indices are epistemically indistinguishable for an agent only if she has the same action types available at both of their associated moments. Phrased differently, only if she knows which action types are available for execution. In the literature on knowledge and action this constraint is known as uniformity of available action types [26–28] and in game theory is usually imposed as a standard requirement (one of the first to suggest this requirement were Herzig and Troquard [25, p. 212, Hypothesis 3]).^27^

Condition (C4) effectively forces epistemic indistinguishability relation from indices onto moments.^28^ Horty and Pacuit [8, pp. 631–632] argue that this way the epistemic agency operator makes most sense: “the (C4) provides an appropriate way of characterising the knowledge of a deliberating agent who is aware that she occupies one of some definite set of moments, may not be sure which, and most important, has not yet decided which action type to execute in light of the available information.”^29^

As a result of these two conditions, Horty and Pacuit’s labelled stit frames comply with the uniformity of available action types and their epistemic indistinguishability relations can be thought of as relations between moments.

#### Coalitions and discrete time

We proceed with the analysis of groups in stit theory. For that purpose we conservatively extend the semantics of labelled stit logic [8] to a group setting, that is, we define several group notions in terms of their individualistic counterpart. In particular, we define group knowledge in terms of individual knowledge, group actions in terms of individual actions, and group action types in terms of individual action types. Following the terminology of epistemic transition systems, we refer to a set of agents $\textit{Ags}$ as the grand coalition, and to any group of agents $\textit{C}\subseteq \textit{Ags}$ as a coalition.

##### Definition 11

(Actions and Knowledge of Coalitions). Let $\mathcal {M}=\langle M$, *H*, <, $\textit{Ags}$, $(\textit{Act}_i^m)$, $(\sim _i)$, $\textit{Tps}$, $\textit{Lbl}$, $\textit{Exn}$, $\pi \rangle$ be a labelled stit model.

- For each coalition $\textit{C}\subseteq \textit{Ags}$, we define
  $$\begin{aligned}\sim _{\textit{C}}\,:=\,\bigcap _{i\in \textit{C}} \sim _i.\end{aligned}$$
  Specifically, for the empty coalition we get $\sim _\emptyset =\textit{Ind}\times \textit{Ind}$. Relation $\sim _{\textit{C}}$ characterizes *distributed knowledge* of coalition $\textit{C}$.
- For each coalition $\textit{C}\subseteq \textit{Ags}$, each moment $m\in M$, and each history $h\in H_m$, we define
  IP $$\begin{aligned} \textit{Act}_{\textit{C}}^m(h):=\bigcap _{i \in \textit{C}} \textit{Act}_i^m(h). \end{aligned}$$
  Specifically, for the empty coalition we get $\textit{Act}_\emptyset ^m(h)=H_m$. It is straightforward to show that $\textit{Act}_\textit{C}^m$ constitutes a partition of $H_m$, and we interpret the cells of such partition as the actions available to coalition $\textit{C}$ at moment *m*.
- For each coalition $\textit{C}\subseteq \textit{Ags}$, each moment $m\in M$, and each history $h\in H_m$, we define  Specifically, for the empty coalition we get . We interpret the tuple ${_\textit{C}(m/h)}$ as the action type that coalition $\textit{C}$ executes at index *m*/*h*.^30^

The following observation expresses that in labelled stit models, at a given moment, each action token corresponds to exactly one action type, and vice versa, each action type corresponds to exactly one action token.

##### Observation 1

Let $\mathcal {M}$ be a labelled stit model and let $\textit{C}\subseteq \textit{Ags}$ be a coalition. For every $m\in M$ and every $h,h'\in H_m$, we have that

Next, we proceed with discussing the restriction to *discrete* labelled stit models. We restrict the frames of labelled stit models to only those where the ordering < is discrete. The requirement of a discrete frame plays an important role in the correspondence between epistemic transition systems and labelled stit models, because only in discrete frames we can simulate a transition as a step from one moment where an action takes place to the next moment where that action’s effect takes place (this requirement was also imposed, for instance, in [25, p. 210, Hypothesis 1]).

##### Definition 12

(Discrete Labelled Stit Models). We say that a labelled stit model $\mathcal {M}$ is *discrete* if and only if the frame it is based on meets the following condition:

- For every $m\in M$ and $h\in H_m$, there exists a unique moment $m^{+h}\in h$ such that $m< m^{+h}$, and there is no moment $m''\in h$ such that $m< m''< m^{+h}$.

In relation to a given index *m*/*h* we call this unique moment $m^{+h}$ the *next moment* and use it to define a successor function.

##### Definition 13

(Successor). Let $\mathcal {M}$ be a discrete labelled stit model. We define a *successor function* on indices $m/h\in \textit{Ind}$ as follows:

$$\begin{aligned} \textit{succ}(m/h):= m^{+h}/h. \end{aligned}$$

Discrete labelled stit models are designed to represent discrete time. This setting is essential in order to define the (temporal) next operator $\textsf{X}$. Formula $\textsf{X}\varphi$ is to be read as saying that $\varphi$ holds next. Finally, we can define *discrete labelled group stit language* and the associated set of evaluation rules involving the operators for coalitions and the next operator.

##### Definition 14

(Full Syntax). The language $\mathcal {L}_{\textsf {GX.kstit}}$ is defined as follows:

$$\begin{aligned} \varphi \,\,{::=}\ p\mid \lnot \varphi \mid (\varphi \wedge \varphi )\mid \Box \varphi \mid [\textit{C}\,\textsf{stit}]\varphi \mid \textsf{K}_\textit{C}\varphi \mid [\textit{C}\,\textsf{kstit}]\varphi \mid \textsf{X}\varphi , \end{aligned}$$

where *p* ranges over a given countable set of propositional variables $\mathcal {P}$, and $\textit{C}$ ranges over the powerset of a finite set of agents $\textit{Ags}$. We define $\Diamond \varphi$ as $\lnot \Box \lnot \varphi$, and similarly, $\langle \textit{C}\,\textsf{stit}\rangle \varphi$ as $\lnot [\textit{C}\,\textsf{stit}]\lnot \varphi$.

##### Definition 15

(Full Evaluation Rules). Let $\mathcal {M}$ be a discrete labelled stit model, let *m*/*h* be an index, let $\textit{C}\subseteq \textit{Ags}$ be a coalition, and let $\varphi \in \mathcal {L}_{\textsf {GX.kstit}}$ be a formula. Then the truth of $\varphi$ at *m*/*h* in $\mathcal {M}$ is given by the following (suppressing the standard propositional clauses and the model $\mathcal {M}$):^31^

We use the abbreviation GX.kstit to denote discrete labelled group stit logic,^32^ that is, the logic composed of discrete labelled group stit language, the class of discrete labelled stit models, and the set of evaluation rules defined above.^33^ We often refer to the components of this logic as the GX.kstit language and a GX.kstit model, respectively.

**Fig. 3:**
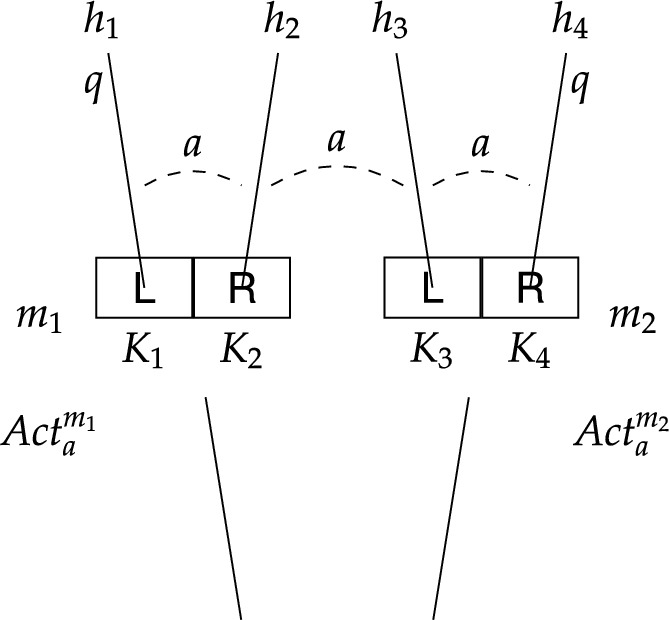
Labelled stit model depicting causal ability to *q* at $m_1$

**Fig. 4:**
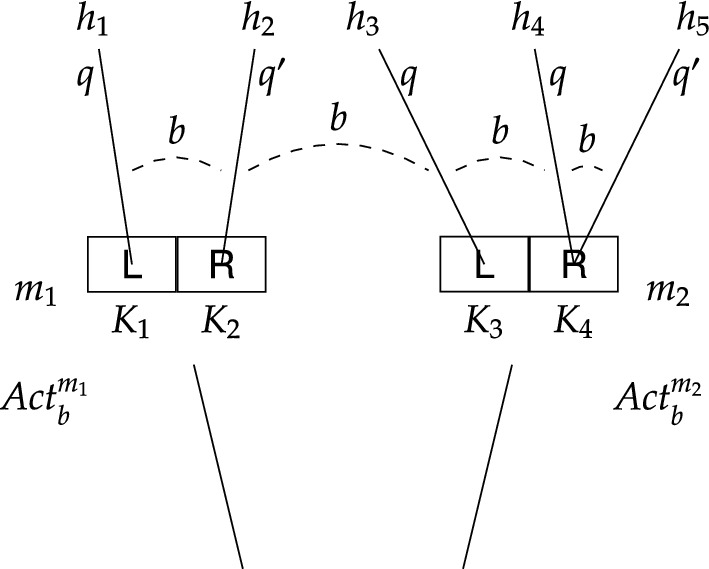
Labelled stit model depicting epistemic ability to *q* at $m_1$ and non-determinism of $K_4$ of type $\textsf {R}$ at $m_2$

Finally, to illustrate how labelled stit logic works, we return to our running example about Alice who is trying to regain control of her Beeble account. More specifically, we introduce two models – one capturing causal ability, and the other capturing epistemic ability and non-determinism. These labelled stit models are depicted in Figs. 3 and 4, respectively (compare to the epistemic transition systems in Figs. 1 and 2, respectively). For a general description let us first concentrate on Fig. 3. There are two moments $m_1$ and $m_2$ and four histories $h_1-h_4$.^34^ Depending on the state of affairs, the agent is either at $m_1$ or $m_2$ and at each of these moments she has two actions available. Each action is identified with a subset of possible histories, in particular, $\textit{Act}_a^{m_1}=\{K_1, K_2\}=\{\{h_1\}, \{h_2\}\}$ and $\textit{Act}_a^{m_2}=\{K_3, K_4\}=\{\{h_3\}, \{h_4\}\}$. While $K_1-K_4$ represent action tokens, $\textsf {L}$ and $\textsf {R}$ represent action types assigned to these action tokens, in particular, $\textit{Lbl}_a(K_1)=\textit{Lbl}_a(K_3)=\textsf {L}$ and $\textit{Lbl}_a(K_2) = \textit{Lbl}_a(K_4) = \textsf {R}$. The agent can either use her laptop, by selecting $\textsf {L}$ represented by $K_1$ at $m_1$ and $K_3$ at $m_2$, or she can use her remote desktop computer, by selecting $\textsf {R}$ represented by $K_2$ at $m_1$ and $K_4$ at $m_2$. The fact that a formula holds at an index *m*/*h* is depicted by that formula being written next to the history *h* as it emanates from the moment *m*. We let *q* denote the state of affairs where the agent regains control of her account, and we see that *q* holds at $m_1/h_1$ and $m_2/h_4$, and is false at $m_1/h_2$ and $m_2/h_3$. The epistemic indistinguishability relation for agent *a* is illustrated by a dashed line between indices with label *a* specifying the agent.^35^ The fact that Alice does not know which device is the trusted one together with the condition (C4) result in one equivalence class consisting of all the indices, that is, the set $\{m_1/h_1, m_1/h_2, m_2/h_3, m_2/h_4\}$.

The labelled stit model in Fig. 3 represents Alice’s ability as desired. It is easy to see that $m_1/h_1\models [{a\,\textsf{stit}}]q$, because *q* is guaranteed at $m_1$ by Alice’s action $K_1$ of type $\textsf {L}$. Effectively, by performing $K_1$ at $m_1$ Alice constrains the possible histories to only those where *q* holds. Similarly, we have that $m_2/h_4\models [{a\,\textsf{stit}}]q$, by performing $K_4$ of type $\textsf {R}$. It follows that $\Diamond [{a\,\textsf{stit}}]q$ holds at any index that is based on moment $m_1$ or $m_2$. Hence, according to this model, Alice is able, in the causal sense, to regain control of her account. As for her epistemic ability, however, we can see that $m_1/h_1\not \models [a\,\textsf{kstit}]q$, because Alice cannot epistemically distinguish $m_1/h_1$ from $m_2/h_3$, where she also executes the action of type $\textsf {L}$ and where *q* does not hold. Also, trivially we have that $m_1/h_2\not \models [a\,\textsf{kstit}]q$. Hence, $\Diamond [a\,\textsf{kstit}]q$ does not obtain and Alice is unable, in the epistemic sense, to regain control of her account.

Now let us look at a slightly different model depicted in Fig. 4 (for the current purpose ignore proposition $q'$ satisfied at indices $m_1/h_2$ and $m_2/h_5$). While the model in Fig. 3 was used to illustrate causal ability, this model is used to illustrate epistemic ability and non-determinism. The structural similarity to the former model is that all indices belong to the same equivalence class, however, there are two important distinctions, both concerning moment $m_2$: (i) action $K_3$ guarantees *q*, and (ii) action $K_4$ might but does not have to result in *q*. The latter is depicted by two histories passing through the R-cell at $m_2$, one satisfying *q*, the other not. Conceptually, this model might represent, as in the case of the ETS in Fig. 2, a situation similar to Alice’s, where her colleague Brox has also had his Beeble account hacked, but unlike Alice, he knows that the trusted device is his laptop. In addition, Brox’ wifi card sometimes malfunctions, and when that happens (moment $m_2$) using the remote desktop computer (action of type $\textsf {R}$) might also result in success (proposition *q*). Brox knows about this sporadic malfunction, but there is no way for him to recognise the current status of his wifi card; more specifically, Brox cannot distinguish between the indices emanating from moment $m_1$, representing scenarios where his wifi card works fine, and the indices emanating from moment $m_2$, representing scenarios where his wifi card malfunctions.

As for Brox’ epistemic ability, it is straightforward to confirm in Fig. 4 that he is epistemically able to regain control of his account. Notice that at both moments $m_1$ and $m_2$ using his laptop (action of type $\textsf {L}$) guarantees *q*. Thus, it is easy to see that $m_1/h_1\models [b\,\textsf{kstit}]q$, because *q* holds at any index that is epistemically indistinguishable from $m_1/h_1$ and where Brox also executes the action of type $\textsf {L}$. By similar reasoning, $m_2/h_3\models [b\,\textsf{kstit}]q$. It follows that $\Diamond [b\,\textsf{kstit}]q$ holds at any index that is based on moment $m_1$ or $m_2$ (obviously, this further implies that $\Diamond [b\,\textsf{stit}]q$ also holds at any index that is based on moment $m_1$ or $m_2$). This means that, unlike Alice (Fig. 3), Brox is able, in the epistemic sense, to regain control of his account.

Stit models, similarly as epistemic transition systems, can capture non-deterministic actions. Let us return to the model in Fig. 4 (compare to the epistemic transition system in Fig. 2). Notice that there are two histories within the $\textsf {R}$-cell at $m_2$, in particular, action $K_4=\{h_4, h_5\}$. Since $q'$ is satisfied at $m_2/h_5$ but not at $m_2/h_4$, it follows that $K_4$ does not guarantee $q'$. Hence, $m_2/h_4\not \models [b\,\textsf{stit}]q'$ as well as $m_2/h_5\not \models [b\,\textsf{stit}]q'$. Moreover, action $K_3$ does not guarantee $q'$ either, and so $m_2/h_3\not \models [b\,\textsf{stit}]q'$. This entails that $\Diamond [b\,\textsf{stit}]q'$ does not obtain at $m_2$, which means that at $m_2$ Brox is unable, even in the causal sense, to see to it that $q'$ holds.

## Transformation and correspondence

Our central correspondence result concerns a comparison between epistemic transition logic [7] and discrete labelled group stit logic (based on labelled stit logic [8]) that involves both the languages and the structures. In this section we first introduce a mapping from epistemic transition language to discrete labelled group stit language (3.1). Then, we provide a systematic transformation of a given epistemic transition system to a discrete labelled stit model (3.2). Finally, based on these frameworks, we show our general correspondence result: the analysis of knowing how and the analysis of (one-step) strategic ability in epistemic transition systems correspond to that of epistemic ability and that of causal ability, respectively, in discrete labelled stit models (3.3).

### Translation

We start by defining a mapping from epistemic transition language to discrete labelled group stit language. The central focus falls on the interpretation of the ETL formulas $\textsf{S}_\textit{C}\varphi$ and $\textsf{H}_\textit{C}\varphi$ to the GX.kstit formulas of the form $\Diamond [\textit{C}\,\textsf{stit}]\textsf{X}\varphi '$ and $\Diamond [\textit{C}\,\textsf{kstit}]\textsf{X}\varphi '$, respectively. Phrased less formally, strategic ability in the ETL language is translated to *the possibility to see to it that* in the GX.kstit language, and know-how is translated to *the possibility to see to it, in the epistemic sense, that*.^36^

#### Definition 16

(Formula Translation). Let $p\in \mathcal {P}$ be a propositional variable, let $\varphi ,\psi \in \mathcal {L}_{\textsf {ETL}}$ be some ETL formulas, and let $\textit{C}\subseteq \textit{Ags}$ be a coalition. The *formula translation* from the ETL language to the GX.kstit language is the function $\textit{Tr}: \mathcal {L}_{\textsf {ETL}}\rightarrow \mathcal {L}_{\textsf {GX.kstit}}$ defined recursively as follows:

$$\begin{aligned} \textit{Tr}(p)&\,\,{:=}\,\, p,\\ \textit{Tr}(\lnot \varphi )&\,\,{:=}\,\, \lnot \textit{Tr}(\varphi ),\\ \textit{Tr}(\varphi \wedge \psi )&\,\,{:=}\,\, \textit{Tr}(\varphi )\wedge \textit{Tr}(\psi ),\\ \textit{Tr}(\textsf{K}_\textit{C}\varphi )&\,\,{:=}\,\,\textsf{K}_\textit{C}\textit{Tr}(\varphi ),\\ \textit{Tr}(\textsf{S}_\textit{C}\varphi )&\,\,{:=}\,\,\Diamond [\textit{C}\,\textsf{stit}]\textsf{X}(\textit{Tr}(\varphi )),\\ \textit{Tr}(\textsf{H}_\textit{C}\varphi )&\,\,{:=}\,\,\Diamond [\textit{C}\,\textsf{kstit}]\textsf{X}(\textit{Tr}(\varphi )). \end{aligned}$$

The translation provides a mapping of the ETL formulas into the GX.kstit formulas that is injective but not surjective. In particular, the ETL (one-step) strategic ability formulas and the ETL know-how formulas are mapped to the fixed combinations of the GX.kstit operators, which implies that only a fragment of the GX.kstit language is used.

### Transformation

Given an epistemic transition system, we define a transform model using unravelling, a well-known technique from modal model theory. Typically, unravelling an epistemic transition system at a given state results in a tree-like model with that state as the root. In our approach, however, unravelling the entire ETS results in a forest of trees, each rooted in one of the original ETS states. Although the idea of unravelling is not that complicated, the technical definition is complex, because one needs to do a fair amount of bookkeeping.

At first we introduce three simple auxiliary functions. Recall that in an ETS, $\textit{W}$ is a set of states, $V^\textit{Ags}$ is a set of action profiles of the grand coalition, and $\mathcal {T}\subseteq \textit{W}\times V^\textit{Ags}\times \textit{W}$ is a set of transitions (Definition 1). Then functions $\textit{begin}$ and $\textit{end}$ take a transition and yield the state in which that transition begins or ends, respectively. Function $\textit{via}$ takes a transition and yields the action profile involved in that transition.

#### Definition 17

(Auxiliary Functions). Let $\mathcal {N}=\langle \textit{W}$, $\textit{Ags}$, $(\sim _i)$, *V*, $\mathcal {T}$, $\pi \rangle$ be an epistemic transition system. Then we define functions $\textit{begin}$, $\textit{end}$: $\mathcal {T}\rightarrow \textit{W}$ and $\textit{via}$: $\mathcal {T}\rightarrow V^\textit{Ags}$ as follows:

$$\begin{aligned} \textit{begin}:(w,\textbf{s},w')&\mapsto w,\\ \textit{end}:(w,\textbf{s},w')&\mapsto w',\\ \textit{via}:(w,\textbf{s},w')&\mapsto \textbf{s}. \end{aligned}$$

In order to define our model transformation we proceed in two steps. Given an epistemic transition system, we first define the transform branching-time structure and, then, define the transform model.

#### Definition 18

(Transform Branching-time Structure). Let $\mathcal {N}=\langle \textit{W}$, $\textit{Ags}$, $(\sim _i)$, *V*, $\mathcal {T}$, $\pi \rangle$ be an epistemic transition system. Then we define the *transform branching-time structure* $\mathcal {M}^{\text {bt}}_{\mathcal {N}}=\langle M,H,<\rangle$ as follows:

1. Because sequences are the building blocks of the transform branching-time structures, we introduce some basic notions regarding sequences: by $|m|$ we denote the number of the elements of the sequence *m*, which is also called the length of the sequence *m*. By $m_{\pmb {k}}$ we denote the $k^{\text {th}}$ element of the sequence *m*, for $k\in \{1,\ldots ,|m|\}$.^37^ We define a set of *moments* $M\subseteq \textit{W}\cup \bigcup \nolimits _{n=1}^{\infty }\mathcal {T}^n$ such that
  $$\begin{aligned} m\in M&\Leftrightarrow m\in \textit{W}\,-\,\text {we call these root moments --, or}\\&\qquad m\text { is a sequence, such that for each }1\le k \le |m|\text { it holds that }\\&\qquad m_{\pmb {k}}\in \mathcal {T}\text { and for each }1\le k<|m|\text { it holds that } \textit{end}(m_{\pmb {k}})=\\&\qquad \textit{begin}(m_{\pmb {k+1}}). \end{aligned}$$
2. For every $m,m'\in M$ we define that $m<m'$ if and only if
  $$\begin{aligned} m\ne m' \text { and } {\left\{ \begin{array}{ll} m=\textit{begin}(m'_{\pmb {1}}) & \quad \text {if } m\in \textit{W},\\ \text {for every } 1\le k\le |m|\text { it holds that } m _{\pmb {k}}=m'_{\pmb {k}} & \quad \text {otherwise.} \end{array}\right. } \end{aligned}$$
3. We define a set of *histories* $H\subseteq 2^{M}$, where each history is a maximal set of linearly ordered moments.

Intuitively this definition says that, first, the set of moments *M* consists of elements in $\textit{W}$ and of finite sequences of transitions of the form $(w_1,\mathbf {s_1},w_2),$ $(w_2,\mathbf {s_2},w_3),$ $(w_3,\mathbf {s_3},w_4), \dots ,$ $(w_{n-1},\mathbf {s_{n-1}},w_n)$. Second, a moment *m* comes before another moment $m'$, if *m* is the initial part of $m'$, that is, if *m* is either the initial state of the first transition of $m'$, or *m* is an initial segment of $m'$. And third, each history is infinite and rooted at a root moment. A history can be thought of as an infinite sequence of transitions, but is technically a root moment plus a countably infinite set of linearly ordered finite sequences of transitions.

A further clarification may be helpful. In ATL, a *path* or a *computation* is typically thought of as an infinite sequence of states (for example, see [14]). In contrast, in our transform branching-time structures, a history can be thought of as an infinite sequence of transitions. The main reason for basing histories on transitions rather than states is the fact that there are epistemic transition systems in which there are multiple different transitions from one state to another.

Before moving to the transform model, we first define another two auxiliary functions on the transform branching-time structures. Function $\textit{nextr}$ takes a moment/history pair and returns the *next transition* along that history following after that moment. Or equivalently, it returns the last transition of the next moment along that history.

To illustrate, consider moment *m* of the form

$$\begin{aligned} (w_1,\mathbf {s_1},w_2),(w_2,\mathbf {s_2},w_3),(w_3,\mathbf {s_3},w_4),\dots ,(w_{|m|},\mathbf {s_{|m|}},w_{|m|+1}) \end{aligned}$$

lying on history *h* of the form

$$\begin{aligned}&~w_1,\\&~\big ((w_1,\mathbf {s_1},w_2)\big ),\\&~\big ((w_1,\mathbf {s_1},w_2),(w_2,\mathbf {s_2},w_3)\big ),\\&~\big ((w_1,\mathbf {s_1},w_2),(w_2,\mathbf {s_2},w_3),(w_3,\mathbf {s_3},w_4)\big ),\\&~\vdots \\&\boxed {\big ((w_1,\mathbf {s_1},w_2),(w_2,\mathbf {s_2},w_3),\dots ,(w_{|m|},\mathbf {s_{|m|}},w_{|m|+1})\big )},\\&~\big ((w_1,\mathbf {s_1},w_2),(w_2,\mathbf {s_2},w_3),\dots ,(w_{|m|},\mathbf {s_{|m|}},w_{|m|+1}),(w_{|m|+1},\mathbf {s_{|m|+1}},w_{|m|+2})\big ),\\&~\vdots \end{aligned}$$

Then $\textit{nextr}(m/h)=(w_{|m|+1},\mathbf {s_{|m|+1}},w_{|m|+2})$. In particular, in case of root moment $w_1$, we get $\textit{nextr}(w_1/h)=(w_1,\mathbf {s_1},w_2)$, because the second moment on *h* is $\big ((w_1,\mathbf {s_1},w_2)\big )$.

Further, function $\textit{state}$ takes a moment/history pair and returns the end state of the last transition of that moment. Or equivalently, it returns the initial state of the next transition along that history following after that moment.

For the purpose of the formal definition of these auxiliary functions, we abuse the sequence notation and specify the following: for any history *h* in a transform branching-time structure, we use its linear order and $k\in \mathbb {Z}^+$, and write $h_{\pmb {k}}$ to denote the $k^\text {th}$ moment of the history *h*. Note that $h_{\pmb {1}}$ is a root moment.

#### Definition 19

(Other Auxiliary Functions). Let $\mathcal {M}^{bt}_\mathcal {N}=\langle M, H, < \rangle$ be a transform branching-time structure. Then we define functions $\textit{nextr}:\textit{Ind}\rightarrow \mathcal {T}$ and $\textit{state}: \textit{Ind}\rightarrow \textit{W}$ as follows:

$$\begin{aligned} \textit{nextr}(m/h)&= {\left\{ \begin{array}{ll} (h_{\pmb {2}})_{\pmb {1}} & \quad \text {if } m\in \textit{W},\\ (h_{\pmb {|m|+2}})_{\pmb {|m|+1}} & \quad \text {otherwise.} \end{array}\right. }\\ \textit{state}(m/h)&=\textit{begin}(\textit{nextr}(m/h)). \end{aligned}$$

Notice that for any two indices emanating from the same moment *m*/*h* and $m/h'$ it holds that $\textit{state}(m/h)=\textit{state}(m/h')$, and so even though in $\textit{state}$ function a history is technically part of the input, the output depends solely on a given moment. We will therefore often suppress the history and write $\textit{state}(m)$ instead of $\textit{state}(m/h)$.

Finally, we can define the transform model.

#### Definition 20

(Transform Model). Let $\mathcal {N}=\langle \textit{W}$, $\textit{Ags}$, $(\sim _i)$, *V*, $\mathcal {T}$, $\pi \rangle$ be an epistemic transition system. Then we define the *transform model* $\mathcal {M}_{\mathcal {N}}=\langle M$, *H*, <, $\textit{Ags}'$, $(\textit{Act}_i^m)$, $(\sim '_i)$, $\textit{Tps}$, $\textit{Lbl}$, $\textit{Exn}$, $\pi '\rangle$ as follows:

1. $\mathcal {M}^{\text {bt}}_{\mathcal {N}}=\langle M,H,<\rangle$ is the transform branching-time structure.
2. $$\textit{Ags}':=\textit{Ags}$$.
3. For every $i\in \textit{Ags}'$ and every $m/h\in \textit{Ind}$ we define
  $$\begin{aligned} \textit{Lbl}_i(m/h):=(\textit{via}(\textit{nextr}(m/h)))_{ {{\pmb {i}}}}. \end{aligned}$$
  As a matter of convention, we use $\textit{Lbl}_\textit{Ags}(m/h)$ to denote the action profile given by $\textit{via}(\textit{nextr}(m/h))$.
4. For every $m/h\in \textit{Ind}$ and every $i\in \textit{Ags}'$ we define
  $$\begin{aligned} \textit{Act}_i^m(h):=\{h'\in H_m\mid \textit{Lbl} _i(m/h')=\textit{Lbl} _i(m/h)\}. \end{aligned}$$
5. For every $m/h, m'/h'\in \textit{Ind}$ and every $i\in \textit{Ags}'$ we define $m/h\sim '_i m'/h'$ if and only if $\textit{state}(m)\sim _i \textit{state}(m')$.
6. $$\textit{Tps}:= V$$.
7. For every $m\in M$, every $i\in \textit{Ags}'$ and every $\tau \in \textit{Tps}$ we define
  $$\begin{aligned} \textit{Exn}_i^m(\tau ):= \{h\in H_m\mid \textit{Lbl} _i(m/h)=\tau \}. \end{aligned}$$
8. For every $m/h\in \textit{Ind}$ we define $\pi '(m/h):= \pi (\textit{state}(m))$.^38^

Noticeably, this construction indicates that the transform model is actually a discrete labelled stit model.

#### Proposition 1

Let $\mathcal {N}$ be an epistemic transition system and let $\mathcal {M}_{\mathcal {N}}$ be its transform model. Then $\mathcal {M}_{\mathcal {N}}$ is a discrete labelled stit model.

#### Proof

Let $\mathcal {N}=\langle \textit{W}$, $\textit{Ags}$, $(\sim _i)$, *V*, $\mathcal {T}$, $\pi \rangle$ be an epistemic transition system and let $\mathcal {M}_{\mathcal {N}}=\langle M$, *H*, <, $\textit{Ags}'$, $(\textit{Act}_i^m)$, $(\sim '_i)$, $\textit{Tps}$, $\textit{Lbl}$, $\textit{Exn}$, $\pi '\rangle$ be its transform model. We need to prove that $\mathcal {M}_\mathcal {N}$ satisfies the constraints stated in Definitions 9 and 12.

In the following, it will be helpful to use the notion of concatenation. For any index *m*/*h*, in case *m* is a sequence, we will write $m\cdot \textit{nextr}(m/h)$ for the usual concatenation of moment *m* with transition $\textit{nextr}(m/h)$; in case *m* is a root moment, we let $m\cdot \textit{nextr}(m/h)$ be the moment $(\textit{nextr}(m/h))$.

- (Branching-time structure) It is clear from item 2 of Definition 18 that < is irreflexive, transitive, and does not have backward branching.
- (Discrete time) Let $m/h\in \textit{Ind}$. From Definition 19, it follows that $m\cdot \textit{nextr}(m/h)$ is a moment on *h*. From Definition 18, it holds that $m<m\cdot \textit{nextr}(m/h)$ and that for every $m'\in h$ such that $m<m'$, $m\cdot \textit{nextr}(m/h)\le m'$. Therefore, from the perspective of Definition 12, we have that $m\cdot \textit{nextr}(m/h)=m^{+h}$.
- (Actions) It is clear from item 4 of Definition 20 that for every $m\in M$ and every $i\in \textit{Ags}'$ we get that $\textit{Act}_i^m$ is a partition of $H_m$. Furthermore, items 3 and 4 of Definition 20 and the fact that $m^{+h}=m\cdot \textit{nextr}(m/h)$ entail that the transform model also satisfies (NC). Items 3, 4 and 6 of Definition 20 and the fact that in epistemic transition systems the set of action profiles of the grand coalition is the Cartesian product of the sets of individual actions available to agents, imply that the transform model satisfies (IA).
- (Indistinguishability) It is clear from item 5 of Definition 20 that for every $i\in \textit{Ags}'$, relation $\sim _i'$ is an epistemic equivalence between indices.
- ($\textit{Lbl}$ and $\textit{Exn}$) Functions $\textit{Lbl}$ and $\textit{Exn}$ are well-defined. Moreover, items 3, 4 and 7 of Definition 20 ensure that the transform model satisfies constraints (EL) and (LE).
- (Valuation) Function $\pi '$ is well-defined.

$$\square $$

We have proved that our transform model is indeed a discrete labelled stit model. The unravelling method applied on epistemic transition systems results in further interesting properties being passed on to these transform GX.kstit models. For instance, due to the uniform domain of actions across all states and all agents in epistemic transition systems, we can observe a corresponding rigorous constraint in the transform GX.kstit models, that is, the uniformity of action types across all moments and all agents.

#### Observation 2

Let $\mathcal {N}$ be an epistemic transition system and let $\mathcal {M}_\mathcal {N}$ be the associated transform discrete labelled stit model. Then for every two agents $i,j\in \textit{Ags}'$ and every two moments $m,m'\in M$ it holds that $\textit{Tps}_i^m= \textit{Tps}_j^{m'}=V$.

This further implies the following:

$$\begin{aligned} \{\textit{Lbl}_\textit{Ags}(m/h)\mid h\in H_m\} = \{\textit{Lbl}_\textit{Ags}(m'/h')\mid h'\in H_{m'}\}=V^\textit{Ags}. \end{aligned}$$

Recall that for their new kstit operator to make sense, Horty and Pacuit [8, p. 628] impose additional constraints (C1) and (C4) on labelled stit frames. It is easy to see that our transform discrete labelled stit models satisfy condition (C1) on the basis of Observation 2, and condition (C4) on the basis of Definitions 19 and 20.

The following proposition specifies another important consequence of the construction of the transform GX.kstit model. It implies that for each moment *m* of the transform GX.kstit model the next transitions emanating from *m* directly correspond to the transitions of the ETS that initiate at $\textit{state}(m)$.

#### Proposition 2

Let $\mathcal {N}$ be an epistemic transition system and let $\mathcal {M}_{\mathcal {N}}$ be the associated transform discrete labelled stit model. Let $w\in \textit{W}$ be a state in $\mathcal {N}$. Then for every $m/h\in \textit{Ind}$ such that $\textit{state}(m/h)=w$, we have that

$$\begin{aligned} \{\textit{nextr}(m/h')\mid h'\in H_m\} = \{\delta \in \mathcal {T} \mid \textit{begin}(\delta )=w\}. \end{aligned}$$

#### Proof

Let $\mathcal {N}=\langle \textit{W}$, $\textit{Ags}$, $(\sim _i)$, *V*, $\mathcal {T}$, $\pi \rangle$ be an epistemic transition system and let $\mathcal {M}_{\mathcal {N}}=\langle M$, *H*, <, $\textit{Ags}'$, $(\textit{Act}_i^m)$, $(\sim '_i)$, $\textit{Tps}$, $\textit{Lbl}$, $\textit{Exn}$, $\pi '\rangle$ be the associated transform discrete labelled stit model. Let *w* be a state in $\mathcal {N}$. Take an arbitrary $m/h\in \textit{Ind}$ such that $\textit{state}(m/h)=w$.

($\subseteq$) Definitions 18 and 19 entail that $\textit{nextr}(m/h)$ is a transition $\delta := (\textit{state}(m/h),$ $\textit{Lbl}_\textit{Ags}(m/h),$ $\textit{state}(\textit{succ}(m/h)))$. Clearly, $\textit{begin}(\delta )=w$.

($\supseteq$) Take an arbitrary transition $\delta \in \mathcal {T}$ such that $\textit{begin}(\delta )=w$. From Definitions 18 and 19 it is clear that concatenation $m\cdot \delta \in M$ (see the proof of Proposition 1 for a precise definition of concatenation). It remains to show that there exists a history $h'\in H_m$ such that $\textit{nextr}(m/h')=\delta$. Let $\Delta :=\delta _1, \delta _2, \delta _3, \ldots$ be a sequence of transitions such that $\delta _1=\delta$, $\textit{via}(\delta _k)=\textit{via}(\delta )$ for every $k\in \mathbb {Z}^+$, and $\textit{end}({\delta _k})=\textit{begin}(\delta _{k+1})$ for every $k\in \mathbb {Z}^+$ – where these transitions are guaranteed to exist, because the action profile $\textit{via}(\delta )$ is executable at every state in $\mathcal {N}$. Consider moments $m''_1, m''_2, m''_3, \ldots$ such that, for every $k\in \mathbb {Z}^+$, $m''_k:= m \cdot (\delta _1, \ldots , \delta _k)$ is the concatenation of *m* and the first *k* transitions of the chosen sequence $\Delta$. Let $h' := \{m'\in M \mid m' \le m\} \cup \{m''_k \mid k\in \mathbb {Z}^+\}$. It follows that $h'\in H_m$ and $\textit{nextr}(m/h')=\delta$, as desired. $\square$

This proposition further entails that for any two indices *m*/*h* and $m'/h'$ such that $\textit{state}(m)=\textit{state}(m')$, there is $h''\in H_{m'}$ such that

$$\begin{aligned} \textit{nextr}(m/h)=\textit{nextr}(m'/h''). \end{aligned}$$

In particular, it means that

$$\begin{aligned} \textit{Lbl}_\textit{Ags}(m/h)&=\textit{Lbl}_\textit{Ags}(m'/h''), \\ \textit{state}(\textit{succ}(m/h)))&=\textit{state}(\textit{succ}(m'/h''). \end{aligned}$$

Given Definitions 11 and 20 and Propositions 1 and 2, there are a couple of interesting points to emphasise. First, any index *m*/*h* in the transform GX.kstit model resembles the state $\textit{state}(m)$ in the ETS in some important ways:

(i) they are propositionally equivalent: $\pi '(m/h) = \pi (\textit{state}(m))$,
(ii) their information sets are similar: for any $m'/h'\in \textit{Ind}$ and any $\textit{C}\subseteq \textit{Ags}$ we have $m/h\sim '_\textit{C}m'/h'$ if and only if $\textit{state}(m)\sim  _C \textit{state}(m')$,
(iii) their transitions are the same: $\{\textit{nextr}(m/h')\mid h'\in H_m\} = \{\delta \in \mathcal {T} \mid \textit{begin}(\delta )=\textit{state}(m)\}$.

It follows directly that in the transform GX.kstit model, being individually similar to their corresponding state, any two indices *m*/*h* and $m'/h'$ that coincide on their state, that is, $\textit{state}(m)=\textit{state}(m')$, are thus also very similar to each other.

Second, in a stit model, histories can be taken to represent the complete temporal evolution of the world. These histories may run infinitely in both the past and the future. However, the unravelling method used in our model transformation (Definition 18) results in a forest of rooted trees (also known as a rooted forest), which is a disjoint union of multiple trees, each with a root moment. Recall that each of these root moments in the transform discrete labelled stit model corresponds to exactly one of the states of the original epistemic transition system. Hence, there are as many trees in the associated transform GX.kstit model as there are states in the ETS. Furthermore, due to the uniform domain of actions across all states and all agents in the original ETS, each history in the transform GX.kstit model runs infinitely to the future, that is, all branches of all the rooted trees are infinite.

**Fig. 5:**
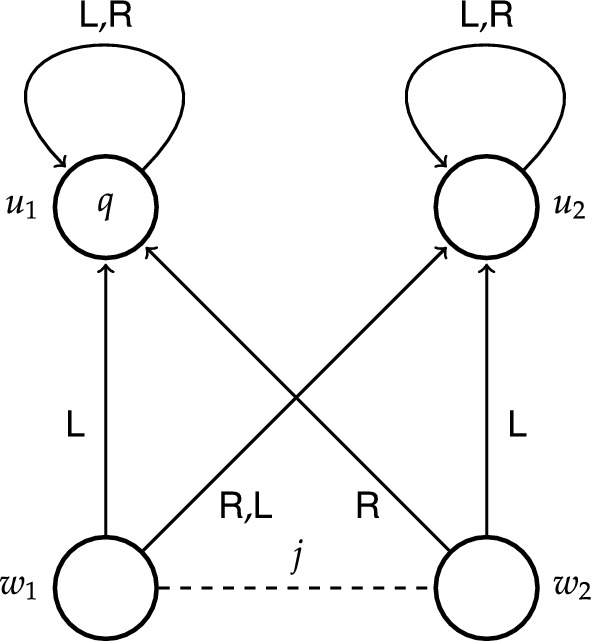
ETS to be transformed into a GX.kstit model

**Fig. 6:**
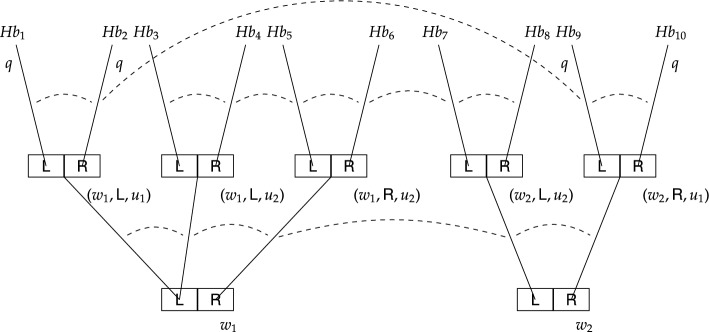
Transform GX.kstit model based on the ETS in Fig. 5

To illustrate the construction of a transform discrete labelled stit model, take the epistemic transition system depicted in Fig. 5, which is inspired by our running example about Alice who is trying to regain control of her Beeble account (instead of Alice, here we have a non-specific agent *j*). Based on this ETS we have constructed the core fragment of its associated transform GX.kstit model and depicted it in Fig. 6. The transform GX.kstit model includes seven moments $w_1, w_2,$ $(w_1,\textsf {L},u_1), (w_1,\textsf {L},u_2),$ $(w_1,\textsf {R},u_2),$ $(w_2,\textsf {L},u_2), (w_2,\textsf {R},u_1)$, and ten history bundles $\textit{Hb}_1$ – $\textit{Hb}_{10}$, each representing a set of histories. For instance, $\textit{Hb}_1$ stands for all histories *h* that run through moment $m:=(w_1, \textsf {L}, u_1)$ and that $\textit{via}(\textit{nextr}(m/h))=\textsf {L}$. Notice that in the original ETS (in Fig. 5) at $w_1$ action $\textsf {L}$ is non-deterministic, leading to $u_1$ as well as $u_2$. In the transform GX.kstit model (in Fig. 6) this is depicted in moment $w_1$ by additional branching of histories within the $\textsf {L}$-cell, that is, some of the histories from the set $\textit{Exn}_j^{w_1}(\textsf {L})$ pass through moment $(w_1, \textsf {L}, u_1)$ and some of them pass through moment $(w_1, \textsf {L}, u_2)$. Let $h_1$ be one of the histories in history bundle $\textit{Hb}_1$. Then $w_1/h_1\not \models [j\,\textsf{stit}]\textsf{X}q$, as action $\textit{Act}_j^{w_1}(h_1)$ allows for the next moment to be $(w_1,\textsf {L},u_2)$, where *q* does not hold. Clearly action $\textit{Exn}_j^{w_1}(\textsf {R})$ does not guarantee $\textsf{X}q$ either. Hence, $\Diamond [j\,\textsf{stit}]\textsf{X}q$ does not hold at any index based on $w_1$. On the contrary, action $\textit{Exn}_j^{w_2}(\textsf {R})$ guarantees $\textsf{X}q$. It follows that $\Diamond [j\,\textsf{stit}]\textsf{X}q$ holds at every index based on $w_2$.

The fact that the agent cannot epistemically distinguish between two indices is depicted by a dashed line between the indices (since there is only one agent in the present case, in Fig. 6 we have dropped the label *j* over the dashed line for better readability).^39^ It is easy to see that in this transform GX.kstit model there are three equivalence classes, corresponding to the epistemic indistinguishability relation of the original ETS, in particular, to the classes $\{w_1,w_2\},\{u_1\},\{u_2\}$.

### Correspondence

#### Lemma 1

Let $\mathcal {N}$ be an epistemic transition system and let $\mathcal {M}_{\mathcal {N}}$ be the associated transform discrete labelled stit model. Let $\varphi \in \mathcal {L}_{\textsf {ETL}}$ be an ETL formula. Then for every state *w* in $\mathcal {N}$ the following are equivalent:

(i) there is an $m/h\in \textit{Ind}$ such that $\textit{state}(m/h)=w$ and $\mathcal {M}_{\mathcal {N}},m/h\models \textit{Tr}(\varphi )$,
(ii) for every $m/h\in \textit{Ind}$ satisfying $\textit{state}(m/h)=w$ it holds that $\mathcal {M}_{\mathcal {N}},m/h\models \textit{Tr}(\varphi )$.

#### Proof

Let $\mathcal {N}=\langle \textit{W}$, $\textit{Ags}$, $(\sim _i)$, *V*, $\mathcal {T}$, $\pi \rangle$ be an epistemic transition system and let $\mathcal {M}_{\mathcal {N}}=\langle M$, *H*, <, $\textit{Ags}'$, $(\textit{Act}_i^m)$, $(\sim '_i)$, $\textit{Tps}$, $\textit{Lbl}$, $\textit{Exn}$, $\pi '\rangle$ be the associated transform discrete labelled stit model. Let $\varphi \in \mathcal {L}_{\textsf {ETL}}$ be an ETL formula.

(ii) $\Rightarrow$ (i) It suffices to prove that for any state $w\in \textit{W}$ there exists an index $m/h\in \textit{Ind}$ such that $\textit{state}(m/h)=w$. Take an arbitrary state $w'\in \textit{W}$ in $\mathcal {N}$. Since $\textit{W}\subseteq M$, $w'$ is a root moment in $\mathcal {M}_\mathcal {N}$. It remains to show that there exists a history that passes through $w'$. We proceed similarly as in the proof of Proposition 2. Take an arbitrary action profile $\mathbf {s'}\in V^\textit{Ags}$. Let $\Delta :=\delta _1, \delta _2, \delta _3, \ldots$ be a sequence of transitions such that $\textit{begin}(\delta _1)=w'$, $\textit{via}(\delta _k)=\mathbf {s'}$ for every $k\in \mathbb {Z}^+$, and $\textit{end}({\delta _k})=\textit{begin}(\delta _{k+1})$ for every $k\in \mathbb {Z}^+$ – where these transitions are guaranteed to exist, because the action profile $\mathbf {s'}$ is executable at every state in $\mathcal {N}$. Consider moments $m'_1, m'_2, m'_3, \ldots$ such that, for every $k\in \mathbb {Z}^+$, $m'_k := (\delta _1, \ldots , \delta _k)$ is the concatenation of the first *k* transitions of the chosen sequence $\Delta$. Let $h' := w' \cup \{m'_k \mid k\in \mathbb {Z}^+\}$. It follows that $h'\in H_{w'}$ and $\textit{state}(w'/h')=w'$, as desired. Hence, for any state *w* in $\mathcal {N}$ there exists an index *m*/*h* in $\mathcal {M}_\mathcal {N}$ such that $\textit{state}(m/h)=w$.

(i) $\Rightarrow$ (ii) We prove this by induction on the complexity of formula $\varphi$. First observe that the propositional cases follow directly from the definition of the valuation $\pi '$ of the transform model $\mathcal {M}_{\mathcal {N}}$ (Definition 20) and the definition of formula translation $\textit{Tr}$ (Definition 16).

- Let $\varphi \equiv \lnot \psi$ and let $w_1$ be a state in $\mathcal {N}$. Let $m_1/h_1$ be an index such that $\textit{state}(m_1/h_1)=w_1$ and $\mathcal {M}_{\mathcal {N}},m_1/h_1\models \textit{Tr}(\lnot \psi )$, or equivalently, $\mathcal {M}_{\mathcal {N}},m_1/h_1\models \lnot \textit{Tr}(\psi )$. It follows that it is not the case that for every $m'/h'$ satisfying $\textit{state}(m'/h')=w_1$ it holds that $\mathcal {M}_{\mathcal {N}},m'/h'\models \textit{Tr}(\psi )$. By induction hypothesis, we get that it is not the case that there exists an $m'/h'$ such that $\textit{state}(m'/h')=w_1$ and $\mathcal {M}_{\mathcal {N}},m'/h'\models \textit{Tr}(\psi )$. Hence, for every $m'/h'$ satisfying $\textit{state}(m'/h')=w_1$ it holds that $\mathcal {M}_{\mathcal {N}},m'/h'\models \lnot \textit{Tr}(\psi )$, as desired.
- The case of $\varphi \equiv \psi _1\wedge \psi _2$ is straightforward.
- Let $\varphi \equiv \textsf{K}_\textit{C}\psi$ and let $w_1$ be a state in $\mathcal {N}$. Let $m_1/h_1$ be an index such that $\textit{state}(m_1/h_1)=w_1$ and $\mathcal {M}_{\mathcal {N}},m_1/h_1\models \textit{Tr}(\textsf{K}_\textit{C}\psi )$, or equivalently, $\mathcal {M}_{\mathcal {N}},m_1/h_1\models \textsf{K}_\textit{C}\textit{Tr}(\psi )$. It follows that for any $m'/h'$ satisfying $m'/h'\sim '_\textit{C}m_1/h_1$ it holds that $\mathcal {M}_{\mathcal {N}},m'/h'\models \textsf{K}_\textit{C}\textit{Tr}(\psi )$. The definition of $\sim '_\textit{C}$ implies that for any $m'/h'$ satisfying $\textit{state}(m'/h')=w_1$ it holds that $m'/h'\sim '_\textit{C}m_1/h_1$. Hence, for every $m'/h'$ satisfying $\textit{state}(m'/h')=w_1$ it holds that $\mathcal {M}_{\mathcal {N}},m'/h'\models \textsf{K}_\textit{C}\textit{Tr}(\psi )$, as desired.
- Let $\varphi \equiv \textsf{S}_\textit{C}\psi$ and let $w_1$ be a state in $\mathcal {N}$. Let $m_1/h_1$ be an index such that $\textit{state}(m_1/h_1)=w_1$ and $\mathcal {M}_{\mathcal {N}},m_1/h_1\models \textit{Tr}(\textsf{S}_\textit{C}\psi )$, or equivalently, $\mathcal {M}_{\mathcal {N}},m_1/h_1\models \Diamond [\textit{C}\,\textsf{stit}]\textsf{X}(\textit{Tr}(\psi ))$. It follows that ($\dagger$) there exists $h_1^*\in H_{m_1}$ such that for every $h'\in \textit{Act}_\textit{C}^{m_1}(h_1^*)$ it holds that $\mathcal {M}_{\mathcal {N}},m_1/h'\models \textsf{X}(\textit{Tr}(\psi ))$, or equivalently, it holds that $\mathcal {M}_{\mathcal {N}},\textit{succ}(m_1/h')\models \textit{Tr}(\psi )$. Take an arbitrary $m_2/h_2\in \textit{Ind}$ such that $\textit{state}(m_2/h_2)=w_1$. We will show that $\mathcal {M}_{\mathcal {N}},m_2/h_2\models$ $\Diamond [\textit{C}\,\textsf{stit}]\textsf{X}(\textit{Tr}(\psi ))$. From Proposition 2 we know that there exists an $h_2^*\in H_{m_2}$ such that $\textit{nextr}(m_2/h_2^*)=\textit{nextr}(m_1/h_1^*)$, which implies that . We will prove that $\mathcal {M}_{\mathcal {N}},m_2/h_2^*\models [\textit{C}\,\textsf{stit}]\textsf{X}(\textit{Tr}(\psi ))$, from which the claim that we want to show follows. Take an arbitrary $h_2'\in \textit{Act}_\textit{C}^{m_2}(h_2^*)$. From Proposition 2 we know that there exists an $h'_1\in H_{m_1}$ such that $\textit{nextr}(m_1/h'_1)=\textit{nextr}(m_2/h'_2)$. This implies that ${\pmb {Lbl}}_C(m_1/h_1')={\pmb {Lbl}}_C(m_2/h'_2)$ and that $\textit{state}(\textit{succ}(m_1/h'_1))=\textit{state}(\textit{succ}(m_2/h'_2))$. Further, Observation 1 entails that ${\pmb {Lbl}}_C(m_2/h'_2)={\pmb {Lbl}}_C(m_2/h_2^*)$, from which we have that ${\pmb {Lbl}}_C(m_1/h'_1)={\pmb {Lbl}}_C(m_1/h_1^*)$. Observation 1 then entails that $h'_1\in \textit{Act}_\textit{C}^{m_1}(h_1^*)$. Therefore, assumption ($\dagger$) renders that $\mathcal {M}_{\mathcal {N}},\textit{succ}(m_1/h'_1)\models \textit{Tr}(\psi )$. By induction hypothesis we know that for every *m*/*h* satisfying $\textit{state}(m/h)=\textit{state}(\textit{succ}(m_1/h'_1))$ it holds that $\mathcal {M}_{\mathcal {N}},m/h\models \textit{Tr}(\psi )$. Since $\textit{state}(\textit{succ}(m_1/h'_1))=\textit{state}(\textit{succ}(m_2/h'_2))$, we have that $\mathcal {M}_{\mathcal {N}},\textit{succ}(m_2/h'_2)\models \textit{Tr}(\psi )$, which implies that $\mathcal {M}_{\mathcal {N}},m_2/h'_2\models \textsf{X}(\textit{Tr}(\psi ))$. Since the above holds for every $h_2'\in \textit{Act}_\textit{C}^{m_2}(h_2^*)$, we have shown that $\mathcal {M}_{\mathcal {N}},m_2/h_2^*\models [\textit{C}\,\textsf{stit}]\textsf{X}(\textit{Tr}(\psi ))$, as desired.
- Let $\varphi \equiv \textsf{H}_\textit{C}\psi$ and let $w_1$ be a state in $\mathcal {N}$. Let $m_1/h_1$ be an index such that $\textit{state}(m_1/h_1)=w_1$ and $\mathcal {M}_{\mathcal {N}},m_1/h_1\models \textit{Tr} (\textsf{H}_\textit{C}\psi )$, or equivalently, $\mathcal {M}_{\mathcal {N}},m_1/h_1\models \Diamond [\textit{C}\,\textsf{kstit}]\textsf{X}(\textit{Tr}(\psi ))$. It follows that ($\dagger$) there exists $h_1^*\in H_{m_1}$ such that for every $m'/h'$ satisfying $m'/h'\sim '_\textit{C}m_1/h_1^*$ and for any $m'/h''$ satisfying ${\pmb {Lbl}}_C(m'/h'')={\pmb {Lbl}}_C(m_1/h_1^*)$, it holds that $\mathcal {M}_{\mathcal {N}},m'/h''\models \textsf{X}(\textit{Tr}(\psi ))$. Take an arbitrary $m_2/h_2\in \textit{Ind}$ such that $\textit{state}(m_2/h_2)=w_1$. We will show that $\mathcal {M}_{\mathcal {N}},m_2/h_2\models \Diamond [\textit{C}\,\textsf{kstit}]\textsf{X}(\textit{Tr}(\psi ))$. From Proposition 2 we know that there exists an $h_2^*\in H_{m_2}$ such that $\textit{nextr}(m_2/h_2^*)=\textit{nextr}(m_1/h_1^*)$, which implies that ${\pmb {Lbl}}_C(m_2/h_2^*)={\pmb {Lbl}}_C(m_1/h_1^*)$. We will prove that $\mathcal {M}_{\mathcal {N}},m_2/h_2^*\models [\textit{C}\,\textsf{kstit}]\textsf{X}(\textit{Tr}(\psi ))$, from which the claim that we want to show follows. Take an arbitrary $m_3/h_3\in \textit{Ind}$ such that $m_3/h_3\sim '_\textit{C}m_2/h_2^*$ and take an arbitrary $h_3^*\in H_{m_3}$ such that ${\pmb {Lbl}}_C(m_3/h_3^*)={\pmb {Lbl}}_C(m_2/h_2^*)$. From Definition 19 we get that $\textit{state}(m_2/h_2^*)=\textit{state}(m_2/h_2)=w_1=\textit{state}(m_1/h_1)=\textit{state}(m_1/h_1^*)$. Therefore, we have that $m_2/h_2^*\sim '_\textit{C}m_1/h_1^*$. Transitivity of $\sim '_\textit{C}$ then yields that $m_3/h_3\sim '_\textit{C}m_1/h_1^*$. Recall that $h_3^*$ satisfies ${\pmb {Lbl}}_C(m_3/h_3^*)={\pmb {Lbl}}_C(m_2/h_2^*)={\pmb {Lbl}}_C(m_1/h_1^*)$. Therefore, assumption ($\dagger$) renders that $\mathcal {M}_{\mathcal {N}},m_3/h_3^*\models \textsf{X}(\textit{Tr}(\psi ))$. The above implies that $\mathcal {M}_{\mathcal {N}},m_2/h_2^*\models [\textit{C}\,\textsf{kstit}]\textsf{X}(\textit{Tr}(\psi ))$, as desired.$\square$

To help understand our main result, it may be useful to anticipate that it establishes that in an epistemic transition system a formula $\varphi$ holds at a state *w* if and only if in the associated transform discrete labelled stit model the formula translation $\textit{Tr}(\varphi )$ holds at every index *m*/*h* for which $\textit{state}(m/h)=w$. Hence, in the transform GX.kstit model any indices that coincide on their state will validate the same translated formulas.

#### Theorem 1

Let $\mathcal {N}$ be an epistemic transition system and let $\mathcal {M}_{\mathcal {N}}$ be the associated transform discrete labelled stit model. Let $\varphi \in \mathcal {L}_{\textsf {ETL}}$ be an ETL formula and let *w* be a state in $\mathcal {N}$. Then the following holds:

$$\begin{aligned}&\mathcal {N},w\models \varphi \text { if and only if for every } m/h\in \textit{Ind}\text { satisfying } \textit{state}(m/h)=w \\&\text {it holds that }\mathcal {M}_{\mathcal {N}},m/h\models \textit{Tr}(\varphi ). \end{aligned}$$

#### Proof

Let $\mathcal {N}=\langle \textit{W}$, $\textit{Ags}$, $(\sim _i)$, *V*, $\mathcal {T}$, $\pi \rangle$ be an epistemic transition system and let $\mathcal {M}_{\mathcal {N}}=\langle M$, *H*, <, $\textit{Ags}'$, $(\textit{Act}_i^m)$, $(\sim '_i)$, $\textit{Tps}$, $\textit{Lbl}$, $\textit{Exn}$, $\pi '\rangle$ be the associated transform discrete labelled stit model. Let $\varphi \in \mathcal {L}_{\textsf {ETL}}$ be an ETL formula and let *w* be a state in $\mathcal {N}$.

We prove this theorem by induction on the complexity of formula $\varphi$. First observe that the propositional cases follow directly from the definition of the valuation $\pi '$ of the transform model $\mathcal {M}_{\mathcal {N}}$ (Definition 20) and the definition of the formula translation *Tr* (Definition 16). In particular, $p\in \mathcal {P}$ holds in *w* of $\mathcal {N}$ if and only if $\textit{Tr}(p)$ holds at all *m*/*h* of $\mathcal {M}_{\mathcal {N}}$ satisfying $\textit{state}(m/h)=w$. (Recall that in Lemma 1 we have proved that for each $w\in \textit{W}$ there exists an *m*/*h* such that $\textit{state}(m/h)=w$.)

- ($\Rightarrow$) Let $\varphi \equiv \lnot \psi$ and let $\mathcal {N},w\models \lnot \psi$. We want to show that for every *m*/*h* satisfying $\textit{state}(m/h)=w$ it holds that $\mathcal {M}_{\mathcal {N}},m/h\models \lnot \textit{Tr}(\psi )$. From the assumption it follows that it is not the case that $\mathcal {N},w\models \psi$. By induction hypothesis, we get that it is not the case that for every *m*/*h* satisfying $\textit{state}(m/h)=w$ it holds that $\mathcal {M}_{\mathcal {N}},m/h\models \textit{Tr}(\psi )$. Equivalently, there is an *m*/*h* such that $\textit{state}(m/h)=w$ and $\mathcal {M}_{\mathcal {N}},m/h\models \lnot \textit{Tr}(\psi )$. By Lemma 1, we get that for every *m*/*h* satisfying $\textit{state}(m/h)=w$ it holds that $\mathcal {M}_{\mathcal {N}},m/h\models \lnot \textit{Tr}(\psi )$, as desired. ($\Leftarrow$) Conversely, let $\mathcal {M}_{\mathcal {N}},m/h\models \lnot \textit{Tr}(\psi )$ for every *m*/*h* satisfying $\textit{state}(m/h)=w$. We want to show that $\mathcal {N},w\models \lnot \psi$. From the assumption it follows that it is not the case that for every *m*/*h* satisfying $\textit{state}(m/h)=w$ it holds that $\mathcal {M}_{\mathcal {N}},m/h\models \textit{Tr}(\psi )$. By induction hypothesis, we get that it is not the case that $\mathcal {N},w\models \psi$. Equivalently, it holds that $\mathcal {N},w\models \lnot \psi$, as desired.
- ($\Rightarrow$) Let $\varphi \equiv \psi _1\wedge \psi _2$ and let $\mathcal {N},w\models \psi _1\wedge \psi _2$. We want to show that for every *m*/*h* satisfying $\textit{state}(m/h)=w$ it holds that $\mathcal {M}_{\mathcal {N}},m/h\models \textit{Tr}(\psi _1)\wedge \textit{Tr}(\psi _2)$. From the assumption it follows that $\mathcal {N},w\models \psi _1$ and $\mathcal {N},w\models \psi _2$. By induction hypothesis for each of the formulas $\psi _1$ and $\psi _2$ separately, we get that for every *m*/*h* satisfying $\textit{state}(m/h)=w$ it holds that $\mathcal {M}_{\mathcal {N}},m/h\models \textit{Tr}(\psi _1)$ and $\mathcal {M}_{\mathcal {N}},m/h\models \textit{Tr}(\psi _2)$. Hence, for every *m*/*h* satisfying $\textit{state}(m/h)=w$ it holds that $\mathcal {M}_{\mathcal {N}},m/h\models \textit{Tr}(\psi _1)\wedge \textit{Tr}(\psi _2)$, as desired. ($\Leftarrow$) The converse is straightforward.
- ($\Rightarrow$) Let $\varphi \equiv \textsf{K}_\textit{C}\psi$ and let $\mathcal {N},w\models \textsf{K}_\textit{C}\psi$. We want to show that for every *m*/*h* satisfying $\textit{state}(m/h)=w$ it holds that $\mathcal {M}_{\mathcal {N}},m/h\models \textsf{K}_\textit{C}\textit{Tr}(\psi )$. Take an arbitrary $m_1/h_1\in \textit{Ind}$ such that $\textit{state}(m_1/h_1)=w$ and an arbitrary $m_2/h_2\in \textit{Ind}$ such that $m_2/h_2\sim '_\textit{C}m_1/h_1$. We want to show that $\mathcal {M}_{\mathcal {N}},m_2/h_2\models \textit{Tr}(\psi )$. By definition of $\sim '_\textit{C}$ we get that $\textit{state}(m_2/h_2)\sim _\textit{C}\textit{state}(m_1/h_1)$, or equivalently, that $\textit{state}(m_2/h_2)\sim _\textit{C}w$. From the assumption it follows that for every $w'\in \textit{W}$ such that $w'\sim _\textit{C}w$ it holds that $\mathcal {N},w'\models \psi$, in particular, $\mathcal {N},\textit{state}(m_2/h_2)\models \psi$. By induction hypothesis, we get that $\mathcal {M}_{\mathcal {N}},m_2/h_2\models \textit{Tr}(\psi )$, as desired. ($\Leftarrow$) Conversely, let $\mathcal {M}_{\mathcal {N}},m/h\models \textsf{K}_\textit{C}\textit{Tr}(\psi )$ for every *m*/*h* satisfying $\textit{state}(m/h)=w$. We want to show that $\mathcal {N},w\models \textsf{K}_\textit{C}\psi$. Take an arbitrary $m_1/h_1\in \textit{Ind}$ such that $\textit{state}(m_1/h_1)=w$ and an arbitrary $w_1\in \textit{W}$ such that $w_1\sim _\textit{C}w$. We want to show that $\mathcal {N},w_1\models \psi$. By definition of $\sim '_\textit{C}$ we have that for any $m'/h'$ satisfying $\textit{state}(m'/h')=w_1$ it holds that $m'/h'\sim '_\textit{C}m_1/h_1$. From the assumption it follows that for any $m'/h'$ satisfying $m'/h'\sim '_\textit{C}m_1/h_1$ it holds that $\mathcal {M}_{\mathcal {N}},m'/h'\models \textit{Tr}(\psi )$. Hence, we have that for every $m'/h'$ satisfying $\textit{state}(m'/h')=w_1$ it holds that $\mathcal {M}_{\mathcal {N}},m'/h'\models \textit{Tr}(\psi )$. By induction hypothesis, we get that $\mathcal {N},w_1\models \psi$, as desired.
- ($\Rightarrow$) Let $\varphi \equiv \textsf{S}_\textit{C}\psi$ and let $\mathcal {N},w\models \textsf{S}_\textit{C}\psi$. We want to show that for every *m*/*h* satisfying $\textit{state}(m/h)=w$ it holds that $\mathcal {M}_{\mathcal {N}},m/h\models \Diamond [\textit{C}\,\textsf{stit}]\textsf{X}(\textit{Tr}(\psi ))$. Take an arbitrary $m_1/h_1\in \textit{Ind}$ such that $\textit{state}(m_1/h_1)=w$. From the assumption it follows that ($\dagger$) there is an $\textbf{s}\in V^\textit{Ags}$ such that for every $w'\in \textit{W}$, if $w\overset{\textbf{s}_\textit{C}}{\rightarrow }w'$ then $\mathcal {N},w'\models \psi$. From Proposition 2 we know that there exists an $h'_1\in H_{m_1}$ such that $\textit{via}(\textit{nextr}(m_1/h_1'))=\textbf{s}$, which in turn implies that ${\pmb {Lbl}}_C(m_1/h_1')=\textbf{s}_\textit{C}$. We will prove that $\mathcal {M}_{\mathcal {N}},m_1/h'_1\models [\textit{C}\,\textsf{stit}]\textsf{X}(\textit{Tr}(\psi ))$. Take an arbitrary $h''_1\in \textit{Act}_\textit{C}^{m_1}(h'_1)$. We want to show that $\mathcal {M}_{\mathcal {N}},m_1/h''_1\models \textsf{X}(\textit{Tr}(\psi ))$, or equivalently, that $\mathcal {M}_{\mathcal {N}},\textit{succ}(m_1/h''_1)\models \textit{Tr}(\psi )$. From Definition 19 we know that $\textit{state}(m_1/h''_1)=\textit{state}(m_1/h_1)=w$. From Observation 1 we get that ${\pmb {Lbl}}_C(m_1/h_1'')={\pmb {Lbl}}_C(m_1/h_1')$. Since , we have that $\textit{state}(m_1/h''_1)\overset{\textbf{s}_\textit{C}}{\rightarrow }\textit{state}(\textit{succ}(m_1/h''_1))$. Assumption ($\dagger$) implies that $\mathcal {N},\textit{state}(\textit{succ}(m_1/h''_1))\models \psi$. By induction hypothesis, we get that $\mathcal {M}_{\mathcal {N}},\textit{succ}(m_1/h''_1)\models \textit{Tr}(\psi )$, as desired. In this way, we have shown that $\mathcal {M}_{\mathcal {N}},m_1/h'_1\models [\textit{C}\,\textsf{stit}] \textsf{X}(\textit{Tr}(\psi ))$, and thus that $\mathcal {M}_{\mathcal {N}},m_1/h_1\models \Diamond [\textit{C}\,\textsf{stit}]\textsf{X}(\textit{Tr}(\psi ))$. ($\Leftarrow$) Conversely, let $\mathcal {M}_{\mathcal {N}},m/h\models \Diamond [\textit{C}\,\textsf{stit}]\textsf{X}(\textit{Tr}(\psi ))$ for every *m*/*h* satisfying $\textit{state}(m/h)=w$. We want to show that $\mathcal {N},w\models \textsf{S}_\textit{C}\psi$. In other words, we want to show that there is an action profile $\textbf{s}_\textit{C}\in V^\textit{C}$ such that for every $w'\in \textit{W}$, if $w\overset{\textbf{s}_\textit{C}}{\rightarrow }w'$ then $\mathcal {N},w'\models \psi$. Take an arbitrary $m_1/h_1\in \textit{Ind}$ such that $\textit{state}(m_1/h_1)=w$. From the assumption it follows that ($\dagger$) there exists $h^*_1\in H_{m_1}$ such that for every $h'\in \textit{Act}_\textit{C}^{m_1}(h^*_1)$ it holds that $\mathcal {M}_{\mathcal {N}},m_1/h'\models \textsf{X}(\textit{Tr}(\psi ))$, or equivalently, it holds that $\mathcal {M}_{\mathcal {N}},\textit{succ}(m_1/h')\models \textit{Tr}(\psi )$. We claim that ${\pmb {Lbl}}_C(m_1/h^*_1)$ is the action profile that we are looking for. Take an arbitrary $w_1\in \textit{W}$ such that $w\overset{{\pmb {Lbl}}_C(m_1/h^*_1)}{\rightarrow }w_1$. Hence, there is an $\textbf{s}\in V^\textit{Ags}$ such that $(w,\textbf{s},w_1)\in \mathcal {T}$ and $\textbf{s}_\textit{C}={\pmb {Lbl}}_C(m_1/h^*_1)$. We want to show that $\mathcal {N},w_1\models \psi$. From Proposition 2 we know that there exists an $h'_1\in H_{m_1}$ such that $\textit{nextr}(m_1/h_1')=(w,\textbf{s},w_1)$. In particular, it implies that . Observation 1 further implies that $h'_1\in \textit{Act}_\textit{C}^{m_1}(h^*_1)$, and thus ($\dagger$) renders that $\mathcal {M}_{\mathcal {N}},\textit{succ}(m_1/h'_1)\models \textit{Tr}(\psi )$. This means that $\textit{succ}(m_1/h_1')$ is an index such that $\textit{state}(\textit{succ}(m_1/h_1'))=w_1$ and $\mathcal {M}_{\mathcal {N}},\textit{succ}(m_1/h'_1)\models \textit{Tr}(\psi )$. Lemma 1 implies that for every $m''/h''$ satisfying $\textit{state}(m''/h'')=w_1$, we have that $\mathcal {M}_{\mathcal {N}},m''/h''\models \textit{Tr}(\psi )$. By induction hypothesis we get that $\mathcal {N},w_1\models \psi$, as desired. In this way, we have shown that $\mathcal {N},w\models \textsf{S}_\textit{C}\psi$.
- ($\Rightarrow$) Let $\varphi \equiv \textsf{H}_\textit{C}\psi$ and let $\mathcal {N},w\models \textsf{H}_\textit{C}\psi$. We want to show that for every *m*/*h* satisfying $\textit{state}(m/h)=w$ it holds that $\mathcal {M}_{\mathcal {N}},m/h\models \Diamond [\textit{C}\,\textsf{kstit}]\textsf{X}(\textit{Tr}(\psi ))$. Take an arbitrary $m_1/h_1\in \textit{Ind}$ such that $\textit{state}(m_1/h_1)=w$. From the assumption it follows that ($\dagger$) there is an $\textbf{s}\in V^\textit{Ags}$ such that for every $w', w''\in \textit{W}$, if $w\sim _\textit{C}w'$ and $w'\overset{\textbf{s}_\textit{C}}{\rightarrow }w''$ then $\mathcal {N},w''\models \psi$. From Proposition 2 we know that there exists an $h'_1\in H_{m_1}$ such that $\textit{via}(\textit{nextr}(m_1/h_1'))=\textbf{s}$, which in turn implies that $_\textit{C}(m_1/h_1')=\textbf{s}_\textit{C}$. We will prove that $\mathcal {M}_{\mathcal {N}},m_1/h'_1\models [\textit{C}\,\textsf{kstit}]\textsf{X}(\textit{Tr}(\psi ))$. Take an arbitrary $m_2/h_2\in \textit{Ind}$ such that $m_2/h_2\sim '_\textit{C}m_1/h'_1$ and take an arbitrary $h'_2\in H_{m_2}$ such that $_\textit{C}(m_2/h'_2)= _\textit{C}(m_1/h'_1)=\textbf{s}_\textit{C}$. In particular, $\textit{state}(m_2/h_2')\overset{\textbf{s}_\textit{C}}{\rightarrow }\textit{state}(\textit{succ}(m_2/h_2'))$. We want to show that $\mathcal {M}_{\mathcal {N}},m_2/h'_2\models \textsf{X}(\textit{Tr}(\psi ))$, or equivalently, that $\mathcal {M}_{\mathcal {N}},\textit{succ}(m_2/h'_2)\models \textit{Tr}(\psi )$. From Definition 19 we know that $\textit{state}(m_1/h_1)=\textit{state}(m_1/h'_1)$ and that $\textit{state}(m_2/h_2)=\textit{state}(m_2/h'_2)$. The definition of $\sim _\textit{C}'$ and the assumption that $m_2/h_2\sim '_\textit{C}m_1/h'_1$ imply that $\textit{state}(m_2/h_2)\sim _\textit{C}\textit{state}(m_1/h'_1)$. Hence, we have that $w=\textit{state}(m_1/h_1)\sim _\textit{C}\textit{state}(m_2/h'_2)$. With the fact that $\textit{state}(m_2/h'_2)\overset{\textbf{s}_\textit{C}}{\rightarrow }\textit{state}(\textit{succ}(m_2/h'_2))$, assumption ($\dagger$) renders that $\mathcal {N},\textit{state}(\textit{succ}(m_2/h'_2))\models \psi$. By induction hypothesis we get that $\mathcal {M}_{\mathcal {N}},\textit{succ}(m_2/h'_2)\models \textit{Tr}(\psi )$, as desired. In this way, we have shown that $\mathcal {M}_{\mathcal {N}},m_1/h'_1\models$ $[\textit{C}\,\textsf{kstit}]\textsf{X}(\textit{Tr}(\psi ))$, and thus that $\mathcal {M}_{\mathcal {N}},m_1/h_1\models$ $\Diamond [\textit{C}\,\textsf{kstit}]\textsf{X}(\textit{Tr}(\psi ))$. ($\Leftarrow$) Conversely, suppose that $\mathcal {M}_{\mathcal {N}},m/h\models \Diamond [\textit{C}\,\textsf{kstit}]\textsf{X}(\textit{Tr}(\psi ))$ for every *m*/*h* satisfying $\textit{state}(m/h)=w$. We want to show that $\mathcal {N},w\models \textsf{H}_\textit{C}\psi$. In other words, we want to show that there is an action profile $\textbf{s}_\textit{C}\in V^\textit{C}$ such that for every $w',w''\in \textit{W}$, if $w\sim _\textit{C}w'$ and $w'\overset{\textbf{s}_\textit{C}}{\rightarrow }w''$, then $\mathcal {N},w''\models \psi$. Take an arbitrary $m_1/h_1\in \textit{Ind}$ such that $\textit{state}(m_1/h_1)=w$. From the assumption it follows that ($\dagger$) there exists an $h^*_1\in H_{m_1}$ such that for every $m'/h'$ satisfying $m'/h'\sim '_\textit{C}m_1/h^*_1$ and for any $m'/h''$ satisfying $_\textit{C}(m'/h'')= _\textit{C}(m_1/h^*_1)$, it holds that $\mathcal {M}_{\mathcal {N}},m'/h''\models \textsf{X}(\textit{Tr}(\psi ))$, or equivalently, that $\mathcal {M}_{\mathcal {N}},\textit{succ}(m'/h'')\models \textit{Tr}(\psi )$. We claim that ${{\pmb {Lbl}}}_\textit{C}(m_1/h^*_1)$ is the action profile that we are looking for. Take an arbitrary $w_1\in \textit{W}$ such that $w\sim _\textit{C}w_1$ and take an arbitrary $w_2\in \textit{W}$ such that $w_1\overset{{{\pmb {Lbl}}}_\textit{C}(m_1/h^*_1)}{\rightarrow }w_2$. Hence, there is an $\textbf{s}\in V^\textit{Ags}$ such that $(w_1,\textbf{s},w_2)\in \mathcal {T}$ and $\textbf{s}_\textit{C}={{\pmb {Lbl}}}_\textit{C}(m_1/h^*_1)$. We want to show that $\mathcal {N},w_2\models \psi$. Take an arbitrary $m_2/h_2\in \textit{Ind}$ such that $\textit{state}(m_2/h_2)=w_1$. Definition 19 implies that $\textit{state}(m_1/h^*_1)=\textit{state}(m_1/h_1)=w$. The definition of $\sim '_\textit{C}$ and the assumption that $w_1\sim _\textit{C}w$ further imply that $m_2/h_2\sim '_\textit{C}m_1/h^*_1$. From Proposition 2 we know that there is an $h^*_2\in H_{m_2}$ such that $\textit{nextr}(m_2/h^*_2)=(w_1,\textbf{s},w_2)$. In particular, it implies that $_\textit{C}(m_2/h^*_2) = \textbf{s}_\textit{C}=  _\textit{C}(m_1/h^*_1)$. Since we have that $m_2/h_2\sim '_\textit{C}m_1/h^*_1$ and that $_\textit{C}(m_2/h^*_2)= _\textit{C}(m_1/h^*_1)$, assumption ($\dagger$) implies that $\mathcal {M}_{\mathcal {N}},\textit{succ}(m_2/h^*_2)\models \textit{Tr}(\psi )$. This means that $\textit{succ}(m_2/h^*_2)$ is an index such that $\textit{state}(\textit{succ}(m_2/h^*_2))=w_2$ and $\mathcal {M}_{\mathcal {N}},\textit{succ}(m_2/h^*_2)\models \textit{Tr}(\psi )$. Lemma 1 then implies that for every $m''/h''$ satisfying $\textit{state}(m''/h'')=w_2$, we have that $\mathcal {M}_{\mathcal {N}},m''/h''\models \textit{Tr}(\psi )$. By induction hypothesis we get that $\mathcal {N},w_2\models \psi$, as desired. In this way, we have shown that $\mathcal {N},w\models \textsf{H}_\textit{C}\psi$.

$$\square $$

To summarise, first we have provided a translation from epistemic transition language to discrete labelled group stit language. Then, we have established a systematic transformation of an epistemic transition system to a discrete labelled stit model. Finally, we have proved our general correspondence result for any such pair of an ETS and its associated transform GX.kstit model. Most importantly, this correspondence shows that the analysis of (one-step) strategic ability and the analysis of knowing how in an epistemic transition system can be simulated in the associated transform GX.kstit model by the analysis of causal ability and by the analysis of epistemic ability, respectively.

## Expressing abilities

Theorem 1 establishes a strong relation between the truth of the ETL formulas in epistemic transition systems [7] and the truth of their GX.kstit translations in labelled stit models [8]. In this section we discuss the advantages of such a mapping from the perspective of knowledge representation and reasoning. In particular, we show that, even for the purpose of representing abilities, discrete labelled group stit language is more expressive than epistemic transition language (4.1). This strengthens the candidacy of stit languages for a canonical representation language for agency and responsibility (4.2).

### Expressivity result

We start by showing that the GX.kstit language is *at least as expressive as* the ETL language. This follows from the fact that Theorem 1 says that any statement that is expressible in ETL can be expressed in GX.kstit. To demonstrate this, let us briefly return to our running example about Alice who is trying to regain control of her Beeble account (introduced in Sect. 1). Using epistemic transition logic, we can formalise, for instance, the statement that (*) *an agent has the (one-step) strategic ability to regain control, but she lacks the know-how to do so*. As shown in Sect. 2.1, with *a* representing Alice as the given agent and *q* representing the state of affairs where the agent regains control of her account, in the ETL language statement (*) is represented as follows: 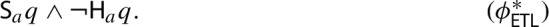 Using our translation from the ETL language to the GX.kstit language (Definition 16), we obtain the following GX.kstit formula: 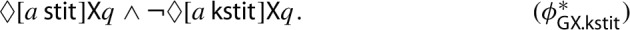 From the perspective of stit theory [8], formula $\phi ^*_{\textsf {GX.kstit}}$ expresses that *the given agent has the causal ability to regain control, but she lacks the epistemic ability to do so*.^40^ It is easy to see that any statement expressible in the ETL language can be expressed in the ETL-fragment of the GX.kstit language. Furthermore, as a corollary of Theorem 1, two states that are mutually distinguishable in ETL correspond to two sets of indices (each consisting of indices coinciding on one of the states) that are mutually distinguishable in GX.kstit.

We proceed by demonstrating that the GX.kstit language is, in fact, *more expressive* than the ETL language; specifically, the former can express more kinds of abilities than the latter. We show that there is a certain kind of ability which is expressible in the former but not in the latter. Consider Zaphod, a colleague of Alice and Brox, who also has had his Beeble account hacked. Like Brox, Zaphod knows that the trusted device is his laptop, and so, Zaphod is epistemically able to regain control of his account. However, unlike both of his colleagues, Zaphod has a third action available; besides using his laptop and using his remote desktop computer, Zaphod can use a medium (that is, Zaphod can delegate the medium to regain control of his account for him). Unbeknownst to Zaphod, the medium is genuine. In particular, Zaphod considers it possible that using the medium guarantees that he regains control of his account, but he also considers it possible that using the medium guarantees that he gets locked out of his account for good. Hence, having the medium available, (**) *Zaphod is able to regain control unknowingly*. Let us refer to the kind of ability in statement (**) as *strict causal ability*.

We formally define strict causal ability in both ETL and GX.kstit.

#### Definition 21

(Strict Causal Ability in ETL). Let $\mathcal {N}$ be an epistemic transition system, let $w\in \textit{W}$ be a state, let $\textit{C}\subseteq \textit{Ags}$ be a coalition, and let $\varphi \in \mathcal {L}_{\textsf {ETL}}$ be a formula. Then we say that coalition $\textit{C}$ is *strictly causally able to* $\varphi$ at $\mathcal {N},w$ if and only if there is an action profile $\textbf{s}_\textit{C}\in V^\textit{C}$ such that

(i) for every $w'\in \textit{W}$: if $w\overset{\textbf{s}_\textit{C}}{\rightarrow }w'$ then $\mathcal {N},w'\models \varphi$; and
(ii) there exist $w'$, $w''\in \textit{W}$ such that $w\sim _\textit{C}w'$, $w'\overset{\textbf{s}_\textit{C}}{\rightarrow }w''$ and $\mathcal {N},w''\not \models \varphi$.

#### Definition 22

(Strict Causal Ability in GX.kstit). Let $\mathcal {M}$ be a discrete labelled stit model, let *m*/*h* be an index, let $\textit{C}\subseteq \textit{Ags}$ be a coalition, and let $\varphi \in \mathcal {L}_{\textsf {GX.kstit}}$ be a formula. Then we say that coalition $\textit{C}$ is *strictly causally able to* $\varphi$ at $\mathcal {M},m/h$ if and only if there is an $h'\in H_m$ such that

(i) for every $h''\in \textit{Act}_\textit{C}^m(h')$ it holds that $\mathcal {M},m/h''\models \varphi$; and
(ii) there exist $m'/h''$, $m'/h'''\in \textit{Ind}$ such that $m/h'\sim _\textit{C}m'/h''$, ${{\pmb {Lbl}}}_\textit{C}(m/h')={{\pmb {Lbl}}}_\textit{C}(m'/h''')$ and $\mathcal {M},m'/h'''\not \models \varphi$.

It should be obvious that the semantic definition of strict causal ability in ETL is similar to the one in GX.kstit. Whenever an agent is strictly causally able to do something, she is (causally) able to unknowingly do it. In contrast, if an agent is not strictly causally able to do something, then she either lacks the causal ability to do it or is only able to knowingly do it.^41^

Observe that strict causal ability follows from statement (*) above expressed by formulas $\phi ^*_\textsf {ETL}$ and $\phi ^*_{\textsf {GX.kstit}}$. However, not the other way around; strict causal ability does not imply statement (*). It is because Zaphod is able to regain control in the strictly causal sense (using the medium), but he is also able to regain control in the epistemic sense (using the laptop). The latter, from the perspective of ETL, corresponds to the statement that Zaphod has the know-how to regain control, which in turn contradicts statement (*). And so, in particular, the ETL formula $\textsf{S}_a q \wedge \lnot \textsf{H}_a q$ does not express strict causal ability.

It is easy to verify that discrete labelled group stit language can express strict causal ability as defined in semantic Definition 22 by the following formula:

$$\begin{aligned} \Diamond ([\textit{C}\,\textsf{stit}]\varphi \wedge \lnot [\textit{C}\,\textsf{kstit}]\varphi ). \end{aligned}$$

Taking *z* to represent Zaphod, we thus formalise the statement (**) as follows: 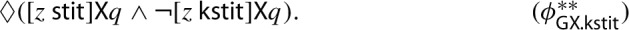 Formula $\phi ^{{*}{*}}_{\textsf {GX.kstit}}$ expresses that it is possible that Zaphod regains control in the causal sense while (by that same action) he does not regain control in the epistemic sense. Hence, it is possible for Zaphod to regain control unknowingly, that is, he has the strict causal ability to regain control.

**Fig. 7:**
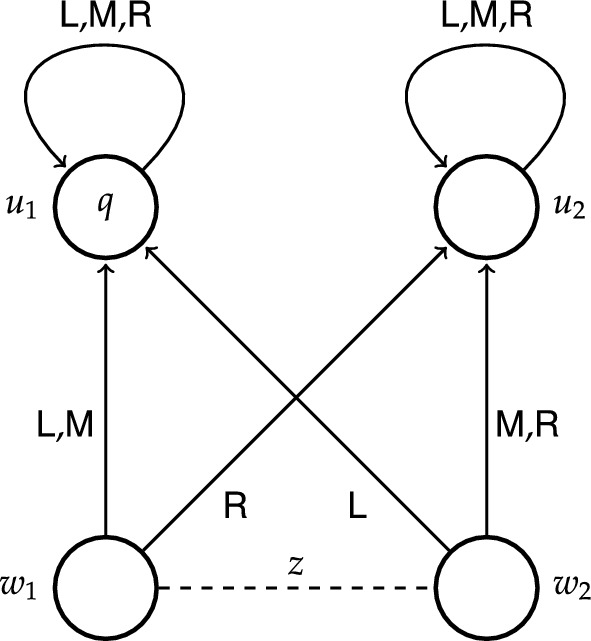
Epistemic transition system $\mathcal {N}$

**Fig. 8:**
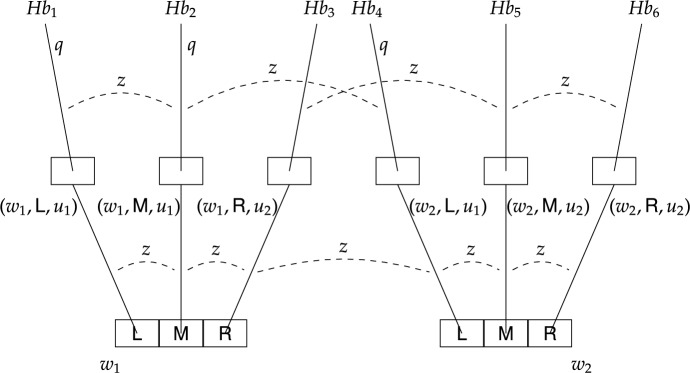
Transform discrete labelled stit model $\mathcal {M}_\mathcal {N}$ based on the epistemic transition system $\mathcal {N}$ in Fig. 7

Although a formula of the form $\textsf{S}_\textit{C}\varphi \wedge \lnot \textsf{H}_\textit{C}\varphi$ does not express strict causal ability, one may ask whether there exists another ETL formula that expresses strict causal ability. The answer is negative: we claim that there is no ETL formula that corresponds to the semantic definition of strict causal ability in Definition 21. We show this using the natural language example of Zaphod, the ETS in Fig. 7, and an induction proof based on this particular example. We start by a brief demonstration that the GX.kstit formula $\phi ^{{*}{*}}_{\textsf {GX.kstit}}$ of the form $\Diamond ([\textit{C}\,\textsf{stit}]\varphi \wedge \lnot [\textit{C}\,\textsf{kstit}]\varphi )$ corresponds to the semantic definition of strict causal ability in Definition 22. We show this using the natural language example of Zaphod and the transform GX.kstit model in Fig. 8.^42^

#### Proposition 3

Unlike discrete labelled group stit language, epistemic transition language cannot express strict causal ability.

#### Proof

Consider the following two models. Figure 7 depicts the epistemic transition system $\mathcal {N}$ and Fig. 8 depicts a core fragment of the associated transform discrete labelled stit model $\mathcal {M}_\mathcal {N}$. Both of them represent Zaphod’s scenario after his Beeble account was hacked. Notice that unlike Alice and Brox, Zaphod has a third action available: besides using his laptop (action L) and using his remote desktop computer (action R), Zaphod can also use a medium (action M). In particular, Zaphod considers it possible that using the medium guarantees that he regains control of his account (at $w_1$), and he also considers it possible that using the medium guarantees that he gets locked out of his account for good (at $w_2$). Importantly, however, states $w_1$ and $w_2$ do not differ in their valuation of propositional variables.

According to our semantic Definitions 21 and 22, in both of these models $\mathcal {N}$ and $\mathcal {M}_\mathcal {N}$ (in Figs. 7 and 8, respectively), Zaphod is strictly causally able to regain control of his account at $w_1$, while he is not strictly causally able to regain control of his account at $w_2$. Hence, if a given language can express strict causal ability, then there must be a formula of that language that distinguishes $w_1$ from $w_2$. Or, equivalently, if $w_1$ and $w_2$ satisfy exactly the same formulas of a given language, then that language cannot express strict causal ability.

Let us start by demonstrating that, on the one hand, the GX.kstit language *can* express strict causal ability. Observe the associated transform discrete labelled stit model in Fig. 8. Let *h* and $h'$ be arbitrary histories such that $h\in \textit{Hb}_1\cup \textit{Hb}_2\cup \textit{Hb}_3$ and $h'\in \textit{Hb}_4\cup \textit{Hb}_5\cup \textit{Hb}_6$.^43^ Then the GX.kstit formula $\phi ^{{*}{*}}_{\textsf {GX.kstit}}$ expressing strict causal ability distinguishes in $\mathcal {M}_\mathcal {N}$ between the indices emanating from moment $w_1$ and the indices emanating from moment $w_2$ (suppressing the model $\mathcal {M}_\mathcal {N}$):^44^

$$\begin{aligned}&w_1/h\models \Diamond ([z\,\textsf{stit}]\textsf{X}q \wedge \lnot [z\,\textsf{kstit}]\textsf{X}q) \quad \text {and}\quad w_2/h'\not \models \Diamond ([z\,\textsf{stit}]\textsf{X}q \wedge \lnot [z\,\textsf{kstit}]\textsf{X}q). \end{aligned}$$

Let us proceed with demonstrating that, on the other hand, the ETL language *cannot* express strict causal ability. Notice that in model $\mathcal {N}$ in Fig. 7 the ETL formula $\textsf{S}_z q\wedge \lnot \textsf{H}_z q$ is false at both $w_1$ and $w_2$, while the formula $\textsf{S}_z q\wedge \textsf{H}_z q$ is true at both. More generally, we show that states $w_1$ and $w_2$ cannot be mutually distinguished by any ETL formula $\varphi$. We prove this claim by induction on the complexity of ETL formula $\varphi$.

First observe that the propositional cases follow directly from the definition of states $w_1$ and $w_2$, as they do not differ in their valuation of propositional variables.

For the induction step, we assume that no subformula of $\varphi$ mutually distinguishes $w_1$ and $w_2$. Then, we proceed by assuming that $\varphi$ holds at $w_1$ and prove that it also holds at $w_2$. The opposite direction is analogous and is thus omitted.

- The case of $\varphi \equiv \lnot \psi$ is straightforward.
- The case of $\varphi \equiv \psi _1\wedge \psi _2$ is straightforward.
- Let $\varphi \equiv \textsf{K}_z\psi$ and let $\mathcal {N},w_1\models \textsf{K}_z\psi$. We want to show that $\mathcal {N},w_2\models \textsf{K}_z\psi$. This is straightforward, because $w_1\sim _z w_2$ and for any $w'\in \textit{W}$ such that $w_1\sim _z w'$ it holds that $\mathcal {N},w'\models \textsf{K}_z\psi$.
- Let $\varphi \equiv \textsf{S}_z\psi$ and let $\mathcal {N},w_1\models \textsf{S}_z\psi$. We want to show that $\mathcal {N},w_2\models \textsf{S}_z\psi$. From the assumption it follows that there is an $\textbf{s}\in V^{\{z\}}$ such that for every $w'\in \textit{W}$, if $w_1\overset{\textbf{s}}{\rightarrow }w'$ then $\mathcal {N},w'\models \psi$. So, either $w_1\overset{\textsf {L}}{\rightarrow }u_1$ (or $w_1\overset{\textsf {M}}{\rightarrow }u_1$) and $\mathcal {N},u_1\models \psi$, or $w_1\overset{\textsf {R}}{\rightarrow }u_2$ and $\mathcal {N},u_2\models \psi$. Hence, from the perspective of state $w_2$, we get either $w_2\overset{\textsf {L}}{\rightarrow }u_1$ and $\mathcal {N},u_1\models \psi$, or $w_2\overset{\textsf {R}}{\rightarrow }u_2$ (or $w_2\overset{\textsf {M}}{\rightarrow }u_2$) and $\mathcal {N},u_2\models \psi$. In either case, we see that $\mathcal {N},w_2\models \textsf{S}_z\psi$, as desired.
- Let $\varphi \equiv \textsf{H}_z\psi$ and let $\mathcal {N},w_1\models \textsf{H}_z\psi$. We want to show that $\mathcal {N},w_2\models \textsf{H}_z\psi$. From the assumption it follows that there is an $\textbf{s}\in V^{\{z\}}$ such that for every $w', w''\in \textit{W}$, if $w_1\sim _z w'$ and $w'\overset{\textbf{s}}{\rightarrow }w''$ then $\mathcal {N},w''\models \psi$. Similarly to the epistemic case of $\textsf{K}_z\psi$, the current case of $\textsf{H}_z\psi$ is straightforward as well: this is because $w_1\sim _z w_2$ and for any $w'\in \textit{W}$ such that $w_1\sim _z w'$ it holds that $\mathcal {N},w'\models \textsf{H}_z\psi$.

In this way, we have shown that in model $\mathcal {N}$ states $w_1$ and $w_2$ correspond on every ETL formula. We can thus conclude that there is no ETL formula that distinguishes $w_1$ and $w_2$, and, therefore, ETL cannot express strict causal ability. $\square$

In particular, this means that there is a certain kind of ability, namely strict causal ability, that is expressible in the GX.kstit language, but not in the ETL language. Hence, formula $\phi ^{{*}{*}}_{\textsf {GX.kstit}}$ is not available in the ETL-fragment of the GX.kstit language.

### Attributing responsibility

We argue that the additional expressivity of discrete labelled group stit language makes it particularly suited to represent two important aspects for attributing responsibility. First, in the philosophical literature on moral responsibility there is a commonly endorsed principle which is called *the principle of alternate possibilities*. This principle states that “a person is morally responsible for what he has done only if he could have done otherwise” [42, p. 829].^45^ Phrased differently, if there was no alternative possibility, the person would not be morally responsible for their action, as the action was unavoidable.

Second, the concept of *modes of mens rea* in legal theory holds that responsibility for an action is directly related to the mental attitude which accompanies the person’s action: purposefully, knowingly, recklessly, negligently, etc. [4]. For our purposes it suffices to recognise that these modes describe different conceptions of agentive engagement with an action; for instance, performing an action strictly causally gives rise to a different kind of responsibility for its outcome than if the action was performed knowingly or intentionally [43].

To formalise the principle of alternate possibilities in the GX.kstit language, we first need to introduce the concept of an agent *refraining from doing*. The concept of refraining, or omitting, was originally characterised by von Wright [44] and then further developed in stit theory [31, 32, 45]: “the idea that an agent *i* refrains from seeing to it that $\varphi$ can be taken to mean that *i* does not see to it that $\varphi$ but that he has the ability to do so” [31, p. 25 – notation adapted]. In discrete labelled group stit logic refraining is expressed by^46^

$$\begin{aligned} \lnot [{i\,\textsf{stit}}]\varphi \wedge \Diamond [{i\,\textsf{stit}}]\varphi . \end{aligned}$$

Then agent *i*’s ability to refrain from seeing to it that $\varphi$ holds (that is, agent *i*’s ability to refrain from $\varphi$-ing) is expressed in GX.kstit by^47^
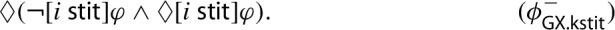


The ability to refrain can also be understood as *the ability to do otherwise*. The GX.kstit language thus provides a straightforward formalisation of the principle of alternate possibilities.

Epistemic transition language can express the ability to refrain as well. Unlike GX.kstit, however, ETL cannot directly express an agent’s ability to not do something. Instead, the ETL language can use the (one-step) strategic ability of the empty coalition and express agent *i*’s ability to refrain from $\varphi$-ing by the formula $\lnot \textsf{S}_\emptyset \varphi \wedge \textsf{S}_i\varphi$. Most interestingly, it turns out that this formula (schematically) corresponds to the aforementioned formula $\phi ^{-}_{\textsf {GX.kstit}}$. This is because in GX.kstit the *agentive* formula $\Diamond \lnot [{i\,\textsf{stit}}]\varphi$, expressing agent *i*’s ability to not see to it that $\varphi$, is in fact equivalent to the *non-agentive* formula $\Diamond \lnot \varphi$, expressing the historical possibility that $\varphi$ does not hold.^48^

The ability to refrain, as the core of the principle of alternate possibilities, can be formalised in both the GX.kstit and the ETL language. However, only the former can do so in a direct (agentive) way, by formalising the concept of not doing and the concept of refraining from doing.

Let us end this section by demonstrating the expressive power of the GX.kstit language for attributing responsibility. The following three exemplary GX.kstit formulas each represent a different combination of a mode of acting and additional information about the abilities for either Alice, or Brox, or Zaphod: 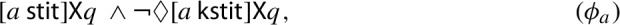

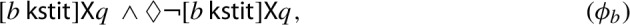

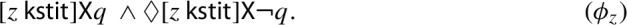


Each of the formulas gives rise to a different notion of responsibility for the action. First, formula $\phi _a$ expresses that Alice sees to it, in the causal sense, that she regains control while she is not able to see to it, in the epistemic sense, that she regains control. Phrased differently, Alice regains control causally and she does not have the ability to do so epistemically. Notice that formula $\phi _a$ is satisfied at indices $m_1/h_1$ and $m_2/h_4$ in the model in Fig. 3.^49^

Second, for the current purposes, let us refer to the formula of the form $\Diamond (\lnot [i\,\,\textsf{kstit}]\varphi \wedge \Diamond [i\,\,\textsf{kstit}]\varphi )$ as agent *i*’s *epistemic* ability to refrain from $\varphi$-ing (compare to the formula $\phi ^{-}_{\textsf {GX.kstit}}$ expressing the ability to refrain). Formula $\phi _b$ expresses that Brox sees to it, in the epistemic sense, that he regains control while he is able to not see to it, in the epistemic sense, that he regains control. It means that Brox regains control epistemically and that he has the epistemic ability to refrain from doing so. Notice that formula $\phi _b$ is satisfied at indices $m_1/h_1$ and $m_2/h_3$ in the model in Fig. 4.^50^ Intuitively, Brox’ responsibility for regaining control is much stronger than Alice’s. This is supported by both the mode of acting and the alternative possibility: Brox’ mode of acting is epistemic while Alice’s is only causal, and Brox has an epistemic alternative possibility while Alice does not.

Third, formula $\phi _z$ expresses that Zaphod sees to it, in the epistemic sense, that he regains control while he is able to see to it, in the epistemic sense, that he does not regain control. Phrased differently, Zaphod regains control epistemically and he has the epistemic ability to prevent it, that is, there is an action available to him by which he would have guaranteed, in the epistemic sense, that he does not regain control. Consider model $\mathcal {M}_\mathcal {N}$ in Fig. 8, and two arbitrary histories $h_1\in \textit{Hb}_1$ and $h_2\in \textit{Hb}_4$. Then formula $\phi _z$ is satisfied at indices $m_1/h_1$ and $m_2/h_2$. Although Brox’ and Zaphod’s mode of acting is the same, their abilities differ: Brox can only refrain from doing the action, but he cannot actually prevent it from happening; Zaphod, on the other hand, can prevent the action from happening.^51^ To distinguish between the ability to refrain and the ability to prevent is essential for responsibility attributions, and the GX.kstit language has a direct (agentive) way to do so.

Unlike the GX.kstit language, the ETL language can express neither actions nor modes of acting, it can only express abilities.^52^ As a consequence, none of the formulas $\phi _a$, $\phi _b$ and $\phi _z$ – nor the different conceptions of responsibility they render – can be expressed in the ETL language.

Let us sum up. We have demonstrated that discrete labelled group stit language is more expressive than epistemic transition language. While the GX.kstit language can express abilities, actions, and modes of acting, the ETL language can express only abilities. However, we have shown that, even for the purpose of representing abilities, the GX.kstit language is more expressive than the ETL language: in particular, there are abilities, such as strict causal ability, that are expressible in the former but not in the latter. In general, as a canonical representation language for agency and responsibility, discrete labelled group stit language is a more suiting candidate than epistemic transition language.

## Conclusion

We have examined two strands of logical frameworks on knowing how and epistemic ability: epistemic transition logic of the ATL family and discrete labelled group stit logic of the stit family, respectively. We have proved a formal correspondence result between two particular instances of these logical paradigms: (i) the analysis of knowing how in epistemic transition systems by Naumov and Tao [7] and (ii) the analysis of epistemic ability in labelled stit models by Horty and Pacuit [8]. What are the implications of this formal result? To explain the implications it is important to recognise that a semantically characterised logical framework consists of three components: a formal language, a class of structures, and a set of compositional evaluation rules.

First, the language translation (Definition 16 and Theorem 1) reveals that the statements about knowing how in epistemic transition language are limited to a subclass of the statements about epistemic ability in discrete labelled group stit language. Similarly, the statements about (one-step) strategic ability in the ETL language are limited to a subclass of the statements about causal ability in the GX.kstit language. In particular, formulas expressing knowing how in the ETL language are mapped to a fixed combination of operators in the GX.kstit language; and the same holds for the ETL formulas expressing (one-step) strategic ability. Moreover, we have shown that the GX.kstit language is more expressive than the ETL language; not only can the former express actions, it can also express more kinds of abilities than the latter.^53^

Second, the model transformation (Definitions 18 and 20, Proposition 1, and Theorem 1) demonstrates that a subclass of discrete labelled stit models can simulate all epistemic transition systems. In other words, the class of discrete labelled stit models is broader than the class of epistemic transition systems. This suggests that GX.kstit models provide a more flexible framework. What do we mean by this flexibility? Suppose we were given an example in natural language. We might be able to figure out whether a particular agent knows how to do something by postulating an ETS of the example and, then, applying the analysis of knowing how to that ETS. Our correspondence result indicates that whenever it is possible to analyse an example using epistemic transition systems, then the same analysis of the example can be carried out using discrete labelled stit models. More succinctly, the analysis of knowing how in epistemic transition systems can be simulated in discrete labelled stit models. It is an open question whether the converse holds: can epistemic transition systems simulate the analysis of epistemic ability in discrete labelled stit models? We believe that it is plausible that there are examples that cannot be analysed using epistemic transition systems – but proving the complete negative conclusion goes beyond the aims of the present paper.

Moreover, given that non-epistemic ATL can be simulated in stit theory [20, 21],^54^ we firmly believe that our correspondence result may be generalised in a similar fashion to the standard treatment of abilities under imperfect information in the ATEL tradition. This would mean that the analysis of ability under uncertainty in ATEL models can be simulated by the analysis of epistemic ability in labelled stit models.

These observations jointly show that it is plausible that stit logics are more expressive and more flexible than epistemic transition logics, but this enhanced expressive power and flexibility may come at a price: while epistemic transition logics [7, 53–55] are often axiomatizable and decidable,^55^ it has been shown that group stit logics are non-axiomatizable and undecidable [59]. However, there are several ways to retrieve axiomatizability, decidability and acceptable complexity results for stit logics [60]. First, we may restrict the formal language. It is well known that stit logics that only include the stit operators for individual agents are often axiomatizable and decidable. Also, Schwarzentruber [61] proves axiomatizability, decidability and complexity results for group stit logics when the coalitions are restricted to a lattice or chains. And, by prohibiting nested stit operators we obtain a group stit logic that is finitely axiomatizable and decidable [46].

Second, we may consider other models to interpret the formal language;^56^ for instance, by weakening the intersection property to the property of monotonic effectivity [21, 26, 28, 60] we obtain a superadditive group stit logic^57^ [63] that is axiomatizable and decidable. In particular, Broersen [43] proves axiomatizability of an xstit logic – a superadditive group stit logic involving operators for actions taking effect in next moments, Payette [64, 65] and Balbiani et al. [66] prove axiomatizability, decidability and complexity results for non-temporal and certain temporal superadditive group stit logics, and Duijf [26, 28] proves axiomatizability of epistemic group stit logic – a superadditive fusion of (non-epistemic) group stit logic and standard epistemic logic. Moreover, we conjecture that non-temporal superadditive group stit logic [66] with distributed knowledge [67] has the finite model property and is thus also decidable.^58^

Third, we may consider other models that give up the independence of agency requirement (see Definition 6, Sect. 2.2.1); the resulting logic coincides with the logic of distributed knowledge which is finitely axiomatizable and decidable [67], and its complexity is in PSPACE [68]. Moreover, epistemic non-independent group stit logic is also axiomatizable – given that it is a fusion of non-epistemic non-independent group stit logic and epistemic logic with distributed and common belief (in line with [26, 28]). Furthermore, we conjecture that non-independent group stit logic with individual and distributed knowledge is also decidable, because non-independent group stit logic and epistemic logic with distributed knowledge both have the finite model property.^59^

In sum, choosing between epistemic transition logics or one of the versions of stit logic discussed above may depend on whether one prioritizes axiomatizability, decidability, or expressitivity.

## Footnotes

1 We appreciate that responsibility is a complicated concept relying not only on ability to prevent but also on other factors such as intention or norms. Although we do not provide a complete analysis of responsibility in this paper, it is one of our central motivations for studying ability.

2 We recognise that the main responsibility falls on the hacker, but that does not necessarily mean that Alice is free of all responsibility.

3 In legal theory, modes of mens rea are typically associated with higher levels of culpability [4].

4 Notice, however, that Alice knew that she had the causal ability to prevent the abuse.

5 To denote the axiomatic system for this logic, Naumov and Tao [7] mostly use the generic term “logical system”, while in the title they call it “an epistemic logic of know-how”. To keep the complex terminology of our paper as clear as possible, we have opted to denote this logic by a name derived from the associated class of structures.

6 For the purpose of our correspondence result, however, we actually use an extension of labelled stit logic. We address the extension in great detail when introducing the framework.

7 Instead of using the term strategic ability, Naumov and Tao [7] use a more descriptive denotation of “having a strategy to transition”.

8 Besides these two families there are also logics of action where particular actions are modelled as individual objects represented by terms in a first-order language, for instance, the event calculus [11].

9 Other well-known hybrids in this respect are alternating-time temporal logic [12] and coalition logic [13]. These two logics differ from each other in their formal languages and in their truth conditions for the central ability operators, but not in their structures, which are concurrent game structures.

10 For the relations between the semantics of coalition logic, epistemic coalition logic, ATL and ATEL see [19].

11 For instance, Broersen, Herzig and Troquard [20, 21] have shown that various analyses of ability in the ATL tradition can be simulated in stit theory.

12 As such epistemic transition systems could be thought of as Kripke models of modal epistemic logic S5 with added labelled transitions. These transitions are taken to be controlled by the agents’ actions.

13 We use bold font for variables that run over a set of action profiles, for instance, $\textbf{s}, \mathbf {s'}, \mathbf {s''}$, or with subscripts $\mathbf {s_1}, \mathbf {s_2}, \mathbf {s_3}$. Notice the difference with $s_1, s_2, s_3$, which denote the actions of individual agents of the action profile $\textbf{s}$.

14 Similar single-agent frameworks in [23, 24] also involve the traditional *skip* action $\epsilon$ such that $(w,\epsilon ,w)\in \mathcal {T}$ for every $w\in \textit{W}$.

15 Herzig and Troquard [25] also suggest to use distributed knowledge. As observed by Naumov and Tao [7] themselves, however, distributed knowledge is not necessarily the most intuitive choice for modeling group knowledge. The classical Byzantine generals problem presents the case where distributed knowledge does not suffice. Instead, *common knowledge* is required.

16 Depending on a context, one could be more explicit and specify the modalities as, for instance, distributed coalition knowledge, coalition (one-step) strategic ability, and distributed coalition know-how, respectively.

17 It is a convention to omit the set notation for singleton coalition subscripts, and thus we write $\textsf{K}_i\varphi$ instead of $\textsf{K}_{\{i\}}\varphi$; similarly for the other two modalities.

18 Specifically for the empty coalition, $\textsf{K}_\emptyset \varphi$ means that formula $\varphi$ must be satisfied at every state of the system, and $\textsf{S}_\emptyset \varphi$ means that formula $\varphi$ is unavoidably satisfied after any single transition [7, pp. 8–9] Additionally, $\textsf{H}_\emptyset \varphi$ means that formula $\varphi$ is satisfied at every state of the system that is an end state of a transition.

19 In the literature on knowledge and action there is a weaker constraint known as *uniformity of available action types* [26–28] and is standardly imposed in game theory. The idea behind this requirement is that whenever two states are indistinguishable for a given agent, then she has the same action types available at these states. In other words, an agent knows which action types are available to her. We discuss the uniformity of available action types in Sect. 2.2.2 (see also [8, 25]).

20 However, there is a well-known folklore trick to model non-determinism in multiagent logics by adding a new fictitious agent such as Environment or Nature.

21 The resulting tuple $\langle M,H,<\rangle$ is called a branching-time frame.

22 See [31, Sect. 2.4] for a useful discussion of the independence of agency requirement.

23 See [33] for an overview of stit theory and its various epistemic extensions.

24 In [34] a similar strategy is pursued by adding *choice names* to epistemic stit models. In particular, Lorini, Longin and Mayor explore responsibility attributions in the context of three different types of epistemic modalities capturing *ex ante* knowledge, *interim* knowledge and *ex post* knowledge, respectively. See also [35] for a Kripke-style semantics for an epistemic stit language with a finite set of *action names*. (We would like to thank an anonymous reviewer for these suggestions.)

25 The execution function is partial, because in general it need not be the case that every action type is available for execution by every agent at every moment. This is in contrast with epistemic transition systems by Naumov and Tao [7].

26 Notice that the (EL) constraint holds only if at a given moment each action type corresponds to exactly one action token. Similarly, the (LE) constraint holds only if each action token corresponds to exactly one action type. Horty and Pacuit [8, p. 627] write: “it seems best to assume that the execution of different action types leads to the performance of different action tokens.” However, that might not be the representation one would want. Consider how many ways there are to prune roses, and how one way of writing the word Afghanistan might be a way to write the word Afghanistan as well as a way to write in general [36].

27 Horty and Pacuit [8, p. 628] recognise that for their new epistemic operator to be well-defined a weaker constraint suffices, namely condition (C2): If $m/h\sim _i m'/h'$, then $\textit{Exn}^{m'}_i(\textit{Lbl} _i(m/h))$ is defined.

28 Although we follow Horty and Pacuit’s [8] modelling decisions, we would like to point out that condition (C4) conflicts with the standard assumption of common knowledge of rationality. Indeed, given reflexivity, it entails that an agent cannot distinguish any moment/history pairs passing through the current moment. In particular, this means that she cannot rule out any moment/history pairs where other agents perform irrational actions. For further epistemic analysis of games see also [37–40]. (We would like to thank an anonymous reviewer for suggesting this clarification.)

29 In general, the standard stit operator is a KT45 modal operator and the new kstit operator is a normal operator satisfying the schema T, but not the schemata 4 and 5. As it turns out, however, within the class of (C4) models, the kstit operator is also a KT45 modal operator.

30 Similarly as in the case of action profiles in epistemic transition systems (see Footnote 13), we use bold font for variables that run over a set of action types for coalitions.

31 Specifically, for the empty coalition, it is easy to see that $[\emptyset \,\textsf{stit}]\varphi \equiv \Box \varphi$, and that $[\emptyset \,\textsf{kstit}]\varphi \equiv \textsf{K}_\emptyset \varphi$, where $\textsf{K}_\emptyset \varphi$ means that formula $\varphi$ must be satisfied at every index of the model.

32 In particular, in the abbreviation G stands for *group*, X stands for *discrete*, and kstit stands for *labelled (epistemic) stit logic*.

33 Notice that coalitions are involved in the language but not in the structures, hence the additional *group* in the name of the language and the logic.

34 Recall that each history represents a complete temporal evolution of the world, and so it is standard in stit models to depict histories as if running from the past through the displayed moments and further to the future.

35 It may be helpful to point out a convention for interpreting the figures: the epistemic indistinguishability relation is closed under reflexivity, transitivity and symmetry.

36 Note that in this context it is assumed that know-how implies (one-step) strategic ability. It means that knowing how to do something implies having the ability to put the know-how in action. Phrased differently, it implies having the power to put the practical knowledge in action. This is, however, a property contested in the literature [41].

37 We use bold font for indexing to emphasize the difference between the indexed elements of sequence *m* such as $m_{\pmb {1}}, m_{\pmb {2}}, m_{\pmb {3}}$, and standardly indexed moments such as $m_1, m_2, m_3$.

38 Given the definition of valuation $\pi '$, it is easy to see that in the transform model $p\leftrightarrow \Box p$ holds for all $p\in \mathcal {P}$.

39 Recall the convention for interpreting the figures: the epistemic indistinguishability relation is closed under reflexivity, transitivity and symmetry.

40 Notice that in Sect. 2.2.3 we effectively use the same conjuncts of formula $\phi ^*_{\textsf {GX.kstit}}$ but without the next operator. The addition of the next operator is the result of the way we design our correspondence between epistemic transition logic and discrete labelled group stit logic. To improve the readability across the two logics, in this section we use the GX.kstit formulas with the next operator (except for the generic formulas).

41 By a simple combination of causal ability and epistemic ability we cannot express the nuances of some interesting situations: for instance, we might want to model a situation like Zaphods’, where it is important to distinguish that despite having an epistemic ability to do something, the agent also has an action available that unbeknownst to her guarantees the same result as the epistemic ability. In such situation the agent has a choice to do something knowingly or to do it unknowingly (to risk it, from her perspective). Or, we might want to model a situation where an agent is not able to do something unknowingly, that is, she either is not able to do it at all or is able to do it knowingly. Each of these situations with respect to its choices might carry different (legal or moral) consequences for an agent, which makes the concept of strict causal ability relevant for responsibility attributions in AI and MAS systems.

42 The natural language example of Zaphod is useful for interpreting the ETS in Fig. 7 and the transform GX.kstit model in Fig. 8. Furthermore, it is useful for explaining the notion of strict causal ability and for explaining the inexpressivity result: there is no ETL formula that expresses some (well-defined) semantic property in epistemic transition systems.

43 For better readability in Fig. 8, we do not display the choice cells and their labels for the moments beyond the level of $w_1$ and $w_2$.

44 In $\mathcal {M}_\mathcal {N}$ indices $w_1/h$ and $w_2/h'$ both satisfy the GX.kstit translation of the ETL formula $\textsf{S}_z q\wedge \textsf{H}_z q$:

$$\begin{aligned}&\mathcal {M}_\mathcal {N},w_1/h\models \Diamond [z\,\textsf{stit}]\textsf{X}q \wedge \Diamond [z\,\textsf{kstit}]\textsf{X}q \quad \text {and}\quad \mathcal {M}_\mathcal {N},w_2/h'\models \Diamond [z\,\textsf{stit}]\textsf{X}q \wedge \Diamond [z\,\textsf{kstit}]\textsf{X}q. \end{aligned}$$

Obviously, (non-strict) causal ability and epistemic ability do not distinguish between the indices emanating from moment $w_1$ and the indices emanating from moment $w_2$.

45 Although Frankfurt [42] explicitly states the principle, he is well-known for arguing against it.

46 Horty [31] introduces the same formula but with the *deliberative* stit operator that was originally proposed by Belnap and Perloff [45] and later simplified by Horty and Belnap [32]: formally, $\lnot [i\,\textsf{dstit}]\varphi \wedge \Diamond [i\,\textsf{dstit}]\varphi$, where $[i\,\textsf{dstit}]\varphi \equiv [{i\,\textsf{stit}}]\varphi \wedge \lnot \Box \varphi$. It is easy to verify that with respect to discrete labelled stit models the two formulas for refraining, each using a different stit operator, are in fact semantically equivalent.

47 Notice that the formula is equivalent to $\Diamond \lnot [{i\,\textsf{stit}}]\varphi \wedge \Diamond [{i\,\textsf{stit}}]\varphi$. This is further equivalent to $\Diamond \langle {i\,\textsf{stit}}\rangle \lnot \varphi \wedge \Diamond [{i\,\textsf{stit}}]\varphi$, which implies agent *i*’s ability to allow for $\lnot \varphi$ to hold. However, that it is not the same as to say that the agent is able to see to it that $\lnot \varphi$ holds.

48 Let us sketch the (schematic) correspondence between the ETL and the GX.kstit formalisation of the ability to refrain. By translating the ETL formula $\lnot \textsf{S}_\emptyset \varphi \wedge \textsf{S}_i\varphi$ to GX.kstit (Definition 16), we get $\lnot \Diamond [\emptyset \,\textsf{stit}]\textsf{X}(\textit{Tr}(\varphi ))\wedge \Diamond [{i\,\textsf{stit}}]\textsf{X}(\textit{Tr}(\varphi ))$. This is equivalent to $\Diamond \lnot \textsf{X}(\textit{Tr}(\varphi ))\wedge \Diamond [{i\,\textsf{stit}}]\textsf{X}(\textit{Tr}(\varphi ))$. By substitution, we get (the non-agentive form) $\Diamond \lnot \varphi '\wedge \Diamond [{i\,\textsf{stit}}]\varphi '$. This is equivalent to (the agentive form) $\Diamond \lnot [{i\,\textsf{stit}}]\varphi '\wedge \Diamond [{i\,\textsf{stit}}]\varphi '$. And finally, this is equivalent to $\Diamond (\lnot [{i\,\textsf{stit}}]\varphi '\wedge \Diamond [{i\,\textsf{stit}}]\varphi ')$, as desired. Moreover, it is easy to verify that formula $\phi ^{-}_{\textsf {GX.kstit}}$ is equivalent to $\Diamond (\lnot \Box \varphi \wedge [{i\,\textsf{stit}}]\varphi )$, which, in the context of deliberative stit, is to be read as saying that agent *i* has the ability to deliberately see to it that $\varphi$. Hence, the ability to refrain from doing something formally coincides with the ability to do it deliberately.

49 We present the current three formulas uniformly with the next operator – see Footnote 40. For the formula to correspond to the model in Fig. 3, we need to omit the next operator, that is, $m_1/h_1\models [a\,\textsf{stit}] q \wedge \lnot \Diamond [a\,\textsf{kstit}] q$. The same holds for $m_2/h_4$.

50 See Footnote 49. For the formula to correspond to the model in Fig. 4, we need to omit the next operator, that is, $m_1/h_1\models [b\,\textsf{kstit}] q \wedge \Diamond \lnot [b\,\textsf{kstit}] q$. The same holds for $m_2/h_3$.

51 The characterisation of responsibility in formula $\phi _z$ comes closest to the very strong sense of responsibility that can be defined by equating responsibility with the ability to prevent [46–49]. Keep in mind, however, that this is just an illustrative example. Responsibility is a complex notion and we do not intend to provide a complete analysis here.

52 Wang [23, p. 8] aptly refers to his transition system by the term *ability map*. The logics of the ATL family allow to reason about abilities (or action possibilities), where the agency behind the abilities is usually not directly expressible; it is expressible what agents *can do*, but not what they *do*. In particular, [23] introduces goal-oriented know-how characterised by a single modality *Kh*. For comparison, in stit logic it is standard to implement syntactically compositional analysis of abilities, such as a combination of impersonal possibility and agency of the form $\Diamond [{i\,\textsf{stit}}]$. However, several extensions of the ATL family with *actions* have been introduced: most relevantly, for a version of ETL with a deliberative epistemic stit operator see [49]; for an extension of ATL with strategic stit operators see [50], for an ATL language with explicit actions see [51, Sect. 4]; and for a stit logic interpreted on concurrent game structures, including a proof of decidability of a discrete-time temporal individual stit logic see [52].

53 We would like to acknowledge that one could consider increasing the expressivity of the epistemic transition language by adding new stit-like operators (see [49] for a version of ETL with a deliberative epistemic stit operator, but without the operators for strategic ability or know-how). One substantial difference would be that the truth conditions of these new stit-like operators require not only states but also transitions or plays, if not whole paths or computations. After all, a state on its own does not determine whether an agent sees to it that $\varphi$ holds. Nevertheless, until it is worked out in detail, it is hard to predict whether such an expanded epistemic transition logic would strike a desirable balance between expressivity and tractability. (We would like to thank an anonymous reviewer for suggesting this option.)

54 In particular, the central coalitional strategic ability operator of ATL expressed by $\langle \langle \textit{C}\rangle \rangle \varphi$, corresponds to the strategic stit formula $\Diamond [\textit{C}\,\textsf{sstit}]\varphi$ (where $[\textit{C}\,\textsf{sstit}]\varphi$ is a *strategic* group stit operator, rather than the stit operators used in this paper).

55 See [23] for a complete axiomatization and decidability of a single-agent logic of goal-directed knowing how, and [24] for a complete axiomatization and decidability of a similar logic including the knowledge modality. For a complete axiomatization and decidability of ATL see [56], and for a complete axiomatization and decidability of epistemic coalition logic with common and distributed knowledge see [57, 58].

56 Lorini, Longin and Mayor [34] prove decidability of their epistemic group stit logics (with choice names) under the assumption that agents’ choices are finite. On a related note, Herzig, Lorini and Perrotin [62] prove that model checking – although generally unfeasible for stit models (because they are typically infinite) – is feasible for finite and compact models. (We would like to thank an anonymous reviewer for these suggestions.)

57 A stit frame $\mathcal {F}=\langle M$, *H*, <, $\textit{Ags}$, $(\textit{Act}_i^m)\rangle$ satisfies the *(full) superadditivity property* if and only if for every coalition $\textit{C}\subseteq \textit{Ags}$ and every index *m*/*h* it holds that $\textit{Act}_\textit{C}^m(h)\subseteq \bigcap _{i\in \textit{C}}\textit{Act}_i^m(h)$. This property is also known as *monotonic effectivity*: if $\textit{C}_1\subseteq \textit{C}_2$ then $\textit{Act}_{\textit{C}_2}^m(h)\subseteq \textit{Act}_{\textit{C}_1}^m(h)$. A stit frame $\mathcal {F}$ satisfies the *(full) additivity property* if and only if for every coalition $\textit{C}\subseteq \textit{Ags}$ and for every index *m*/*h* it holds that $\textit{Act}_\textit{C}^m(h)=\bigcap _{i\in \textit{C}}\textit{Act}_i^m(h)$. This is also known as the *intersection property*. See the (IP) condition of Definition 11, Sect. 2.2.3.

58 We see prospects for combining the respective proof techniques in [66] and [67]; the details will have to be worked out in future work. Furthermore, it would be interesting to investigate whether this conjecture extends to a superadditive version of *labelled* group stit logic and whether the proof technique in [66] can be extended to superadditive epistemic group stit logics and/or to a superadditive version of labelled group stit logic *with the next operator* – even though the original proof technique does not rely on the finite model property.

59 In future work, it may be interesting to investigate whether the conjecture extends to a non-independent version of labelled group stit logic and whether the conjecture extends to temporal versions of these non-independent stit logics.

## References

[1] Matthias, A. (2004). The responsibility gap: Ascribing responsibility for the actions of learning automata. *Ethics and Information Technology,**6*, 175–183.

[2] Moore, M. S. (2009). *Causation and responsibility: An essay in law, morals, and metaphysics*. Oxford: Oxford University Press.

[3] Chopra, S., & White, L. F. (2011). *A legal theory for autonomous artificial agents*. Ann Arbor: University of Michigan Press.

[4] Dubber, M. D. (2002). *Criminal law: Model penal code*. Turning point series. New York: Foundation Press.

[5] Ågotnes, T., Goranko, V., Jamroga, W., & Wooldridge, M. (2015). Knowledge and ability. In H. van Ditmarsch, J. Y. Halpern, W. van der Hoek, & B. Kooi (Eds.), *Handbook of epistemic logic* (pp. 543–589). London: College Publications.

[6] Broersen, J., & Herzig, A. (2015). Using STIT theory to talk about strategies. In J. van Benthem, S. Ghosh, & R. Verbrugge (Eds.), *Models of strategic reasoning* (pp. 137–173). Berlin, Heidelberg: Springer.

[7] Naumov, P., & Tao, J. (2018). Together we know how to achieve: An epistemic logic of know-how. *Artificial Intelligence,**262*, 279–300.

[8] Horty, J. F., & Pacuit, E. (2017). Action types in stit semantics. *The Review of Symbolic Logic,**10*(4), 617–637.

[9] Pratt, V. R. (1976). Semantical considerations on Floyd-Hoare logic. In *Proceedings of the seventeenth symposium on the foundations of computer science* (pp. 109–121). Washington, DC: IEEE Computer Society.

[10] Belnap, N., Perloff, M., & Xu, M. (2001). *Facing the future: Agents and choices in our indeterminist world*. Oxford: Oxford University Press.

[11] Kowalski, R., & Sergot, M. (1986). A logic-based calculus of events. *New Generation Computing,**4*, 67–94.

[12] Alur, R., Henzinger, T. A., & Kupferman, O. (2002). Alternating-time temporal logic. *Journal of the ACM,**49*(5), 672–713.

[13] Pauly, M. (2002). A modal logic for coalitional power in games. *Journal of Logic and Computation,**12*(1), 149–166.

[14] van der Hoek, W., & Wooldridge, M. (2003). Cooperation, knowledge, and time: Alternating-time temporal epistemic logic and its applications. *Studia Logica,**75*(1), 125–157.

[15] Jamroga, W. (2003). Some remarks on alternating temporal epistemic logic. In B. Dunin-Keplicz, & R. Verbrugge (Eds.), *Proceedings of formal approaches to multi-agent systems* (pp. 133–140).

[16] Jamroga, W., & van der Hoek, W. (2004). Agents that know how to play. *Fundamenta Informaticae,**63*(2–3), 185–219.

[17] Schobbens, P.-Y. (2004). Alternating-time logic with imperfect recall. *Electronic Notes in Theoretical Computer Science,**85*, 82–93.

[18] Jamroga, W., & Ågotnes, T. (2007). Constructive knowledge: What agents can achieve under imperfect information. *Journal of Applied Non-Classical Logics,**17*(4), 423–475.

[19] Goranko, V., & Jamroga, W. (2004). Comparing semantics of logics for multi-agent systems. *Synthese,**139*(2), 241–280.

[20] Broersen, J., Herzig, A., & Troquard, N. (2006). Embedding alternating-time temporal logic in strategic logic of agency. *Journal of Logic and Computation,**16*(5), 559–578.

[21] Broersen, J., Herzig, A., & Troquard, N. (2007). A normal simulation of coalition logic and an epistemic extension. In D. Samet (Ed.), *Proceedings of the eleventh conference on theoretical aspects of rationality and knowledge* (pp. 92–101). New York: ACM.

[22] van Benthem, J., & Bergstra, J. A. (1995). Logic of transition systems. *Journal of Logic, Language and Information,**3*(4), 247–283.

[23] Wang, Y. (2018). A logic of goal-directed knowing how. *Synthese,**195*(10), 4419–4439.

[24] Fervari, R., Herzig, A., Li, Y., & Wang, Y. (2017). Strategically knowing how. In C. Sierra (Ed.), *Proceedings of the twenty-sixth international joint conference on artificial intelligence* (pp. 1031–1038).

[25] Herzig, A., & Troquard, N. (2006). Knowing how to play: Uniform choices in logics of agency. In G. Weiss, & P. Stone (Eds.), *Proceedings of the fifth international conference on autonomous agents and multiagent systems* (pp. 209–216). New York: ACM.

[26] Duijf, H. (2018). *Let’s Do It!: Collective responsibility, joint action, and participation*. Dissertation, Utrecht University.

[27] Duijf, H., Broersen, J., Kuncová, A., & Ramírez Abarca, A. I. (2021). Doing without action types. *The Review of Symbolic Logic,**14*(2), 380–410.

[28] Duijf, H. (2022). *The logic of responsibility voids.* Synthese library. Cham: Springer.

[29] Kanger, S. (1971). New foundations for ethical theory. In R. Hilpinen (Ed.), *Deontic logic: Introductory and systematic readings* (pp. 36–58). Dordrecht: Springer.

[30] Pörn, I. (1977). *Action theory and social science: Some formal models*. Dordrecht: Springer.

[31] Horty, J. F. (2001). *Agency and deontic logic*. New York: Oxford University Press.

[32] Horty, J. F., & Belnap, N. (1995). The deliberative stit: A study of action, omission, ability, and obligation. *Journal of Philosophical Logic,**24*(6), 583–644.

[33] Xu, M. (2015). Combinations of stit with ought and know. *Journal of Philosophical Logic,**44*(6), 851–877.

[34] Lorini, E., Longin, D., & Mayor, E. (2014). A logical analysis of responsibility attribution: emotions, individuals and collectives. *Journal of Logic and Computation,**24*(6), 1313–1339.

[35] Herzig, A., & Lorini, E. (2010). A dynamic logic of agency I: STIT, capabilities and powers. *Journal of Logic, Language and Information,**19*, 89–121.

[36] Hornsby, J. (2012). Ryle’s knowing-how, and knowing how to act. In J. Bengson, & M. A. Moffett (Eds.), *Knowing how: Essays on knowledge, mind, and action* (pp. 80–97). New York: Oxford University Press.

[37] de Bruin, B. P. (2004). *Explaining games: On the logic of game theoretic explanations*. Dissertation, University of Amsterdam.

[38] van Benthem, J. (2007). Rational dynamics and epistemic logic in games. *International Game Theory Review,**9*, 13–45.

[39] Bonanno, G. (2008). A syntactic approach to rationality in games with ordinal payoffs. In G. Bonanno, W. van der Hoek, & M. Wooldridge (Eds.), *Logic and the foundations of game and decision theory (LOFT 7)* (pp. 59–86). Amsterdam: Amsterdam University Press.

[40] Lorini, E., & Schwarzentruber, F. (2010). A modal logic of epistemic games. *Games,**1*(4), 478–526.

[41] Alter, T. (2008). Know-how, ability, and the ability hypothesis. *Theoria,**67*(3), 229–239.

[42] Frankfurt, H. G. (1969). Alternate possibilities and moral responsibility. *The Journal of Philosophy,**66*(23), 829–839.

[43] Broersen, J. (2011). Deontic epistemic stit logic distinguishing modes of mens rea. *Journal of Applied Logic,**9*(2), 137–152.

[44] von Wright, G. H. (1963). *Norm and action: A logical enquiry*. New York: Routledge and Kegan Paul.

[45] Belnap, N., & Perloff, M. (1988). Seeing to it that: A canonical form for agentives. *Theoria,**54*(3), 175–199.

[46] Lorini, E., & Schwarzentruber, F. (2011). A logic for reasoning about counterfactual emotions. *Artificial Intelligence,**175*(3–4), 814–847.

[47] Bulling, N., & Dastani, M. (2013). Coalitional responsibility in strategic settings. In J. Leite, T. C. Son, P. Torroni, L. van der Torre, & S. Woltran (Eds.), *Computational logic in multi-agent systems* (pp. 172–189). Berlin, Heidelberg: Springer.

[48] Naumov, P., & Tao, J. (2020). An epistemic logic of blameworthiness. *Artificial Intelligence,**283*, 103269.

[49] Naumov, P., & Tao, J. (2021). Two forms of responsibility in strategic games. In Z.-H. Zhou (Ed.), *Proceedings of the thirtieth international joint conference on artificial intelligence* (pp. 1989–1995).

[50] Broersen, J., Herzig, A., & Troquard, N. (2006). A STIT-extension of ATL. In M. Fisher, W. van der Hoek, B. Konev, & A. Lisitsa (Eds.), *Logics in artificial intelligence* (pp. 69–81). Berlin: Springer.

[51] Ågotnes, T. (2006). Action and knowledge in alternating-time temporal logic. *Synthese,**149*, 375–407.

[52] Boudou, J., & Lorini, E. (2018). Concurrent game structures for temporal STIT logic. In *Proceedings of the seventeenth international conference on autonomous agents and multiagent systems* (pp. 381–389). New York: ACM.

[53] Naumov, P., & Tao, J. (2017). Coalition power in epistemic transition systems. In *Proceedings of the sixteenth international conference on autonomous agents and multiagent systems* (pp. 723–731).

[54] Naumov, P., & Tao, J. (2018). Second-order know-how strategies. In *Proceedings of the seventeenth international conference on autonomous agents and multiagent systems* (pp. 390–398).

[55] Naumov, P., & Tao, J. (2018). Strategic coalitions with perfect recall. In *Proceedings of the thirty-second AAAI conference on artificial intelligence* (pp. 4702–4709).

[56] Goranko, V., & van Drimmelen, G. (2006). Complete axiomatization and decidability of alternating-time temporal logic. *Theoretical Computer Science,**353*, 93–117.

[57] Ågotnes, T., & Alechina, N. (2012). Epistemic coalition logic: Completeness and complexity. In *Proceedings of the eleventh international conference on autonomous agents and multiagent systems* (pp. 1099–1106).

[58] Ågotnes, T., & Alechina, N. (2019). Coalition logic with individual, distributed and common knowledge. *Journal of Logic and Computation,**29*(7), 1041–1069.

[59] Herzig, A., & Schwarzentruber, F. (2008). Properties of logics of individual and group agency. In M. Kracht, M. de Rijke, H. Wansing, & M. Zakharyaschev (Eds.), *Advances in modal logic* (pp. 133–149). Stanford: CSLI Publications.

[60] Van De Putte, F., Tamminga, A., & Duijf, H. (2017). Doing without nature. In J. Seligman, T. Yamada, & A. Baltag (Eds.), *Logic, rationality, and interaction* (pp. 209–223). Berlin, Heidelberg: Springer.

[61] Schwarzentruber, F. (2012). Complexity results of STIT fragments. *Studia Logica,**100*(5), 1001–1045.

[62] Herzig, A., Lorini, E., & Perrotin, E. (2022). A computationally grounded logic of ‘seeing-to-it-that’. In L. De Raedt (Ed.), *Proceedings of the thirty-first international joint conference on artificial intelligence* (pp. 2648–2654).

[63] Ciuni, R., & Lorini, E. (2018). Comparing semantics for temporal STIT logic. *Logique et Analyse,**61*(243), 299–339.

[64] Payette, G. (2012). Completeness of XPstit logic: An extension of Xstit. unpublished.

[65] Payette, G. (2014). Decidability of an Xstit logic. *Studia Logica,**102*(3), 577–607.

[66] Balbiani, P., Gasquet, O., Herzig, A., Schwarzentruber, F., & Troquard, N. (2008). Coalition games over Kripke semantics: Expressiveness and complexity. In C. Dégremont, L. Keiff, & H. Rückert (Eds.), *Dialogues, logics and other strange things. Essays in honour of Shahid Rahman* (pp. 11–32). London: College Publications.

[67] Fagin, R., Halpern, J. Y., Moses, Y., & Vardi, M. Y. (2003). *Reasoning about knowledge*. Cambridge: MIT Press.

[68] Halpern, J. Y., & Moses, Y. (1992). A guide to completeness and complexity for modal logics of knowledge and belief. *Artificial Intelligence,**54*(3), 319–379.

